# Synthesis and In Vitro Evaluation of Caffeoylquinic Acid Derivatives as Potential Hypolipidemic Agents

**DOI:** 10.3390/molecules24050964

**Published:** 2019-03-09

**Authors:** Yu Tian, Xiao-Xue Cao, Hai Shang, Chong-Ming Wu, Xi Zhang, Peng Guo, Xiao-Po Zhang, Xu-Dong Xu

**Affiliations:** 1 Beijing Key Laboratory of Innovative Drug Discovery of Traditional Chinese Medicine (Natural Medicine) and Translational Medicine; Key Laboratory of Bioactive Substances and Resources Utilization of Chinese Herbal Medicine, Ministry of Education; Key Laboratory of Efficacy Evaluation of Chinese Medicine against Glycolipid Metabolic Disorders, State Administration of Traditional Chinese Medicine; Zhong Guan Cun Open Laboratory of the Research and Development of Natural Medicine and Health Products; Key Laboratory of New Drug Discovery Based on Classic Chinese Medicine Prescription; Key Laboratory of New Drug Discovery Based on Classic Chinese Academy of Medical Sciences; Institute of Medicinal Plant Development, Chinese Academy of Medical Sciences & Peking Union Medical College, Beijing 100193, China; ytian@implad.ac.cn (Y.T.); snow2018cxx@163.com (X.-X.C.); hshang@implad.ac.cn (H.S.); cmwu@implad.ac.cn (C.-M.W.); 2 Center of Research and Development on Life Sciences and Environment Sciences, Harbin University of Commerce, Harbin 150076, China; 18800467885@163.com; 3 School of Pharmacy, Hainan Medical University, Haikou 571199, China

**Keywords:** caffeoylquinic acids, chlorogenic acid, derivatives, lipid-lowering effects, oleic acid-elicited, HepG2 cells

## Abstract

A series of novel caffeoylquinic acid derivatives of chlorogenic acid have been designed and synthesized. Biological evaluation indicated that several synthesized derivatives exhibited moderate to good lipid-lowering effects on oleic acid-elicited lipid accumulation in HepG2 liver cells. Particularly, derivatives **3d**, **3g**, **4c** and **4d** exhibited more potential lipid-lowering effect than the positive control simvastatin and chlorogenic acid. Further studies on the mechanism of **3d**, **3g**, **4c** and **4d** revealed that the lipid-lowering effects were related to their regulation of TG levels and merit further investigation.

## 1. Introduction

Hyperlipidemia is among the key risk factors for cardio-vascular diseases (CVD), inducing essential hypertension (EHT), coronary heart disease (CHD), heart failure, atherosclerosis (AS), and sudden cardiac death (SCD). Several studies [1,2,3,4,5] have provided evidence that low-density lipoprotein cholesterol (LDL-c) has been considered as the main risk factor of CHD and AS [6,7], and the increased circulating levels of triglycerides (TG) show a significant influence on CHD and AS [8,9]. Therefore, it is quite beneficial to research and explore anti-hypolipidemic agents that could modulate the dysregulation of lipid metabolism and decrease the elevated levels of serum TG [10].

**Figure 1:**
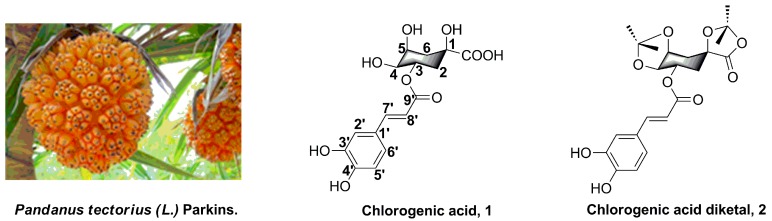
The plant *Pandanus tectorius* (L.) Parkins. and the structures of chlorogenic acid (**1**) and its analogues Chlorogenic acid diketal (**2**).

*Pandanus tectorius* (L.) Parkins. (PTPs, Figure 1), distributed in South China, is a traditional folk medicine for the treatment of leprosy, bronchitis, measles, dermatitis, and diabetes [11,12]. Chlorogenic acid (3-*O*-caffeoylquinic acid, 1, Figure 1) is one of the secondary phenolic metabolites, which is one of the the primary components found in PTPs, and was reported to have potential antioxidant, antimicrobial, anti-inflammatory, and anti-hpyerglycemic effects [13,14,15,16]. In particular, chlorogenic acids and their analogues, which contain one or more caffeoylquinic acid scaffolds, also efficiently improve hypercholesterolemia and hyperglycemia. In addition, as a kind of rich natural product, chlorogenic acids and their analogues are widely distributed in a variety of plants that show hypolipidemic functions, such as *Hypericum salsugineum*, *L. czeczottianus*, *L. nissolia*, *Hypericum androsaemum* L., and *Ipomea batatas* L. [17,18,19,20]. Moreover, our previous studies revealed that some of the natural caffeoylquinic acids were confirmed to possess obvious anti-hyperlipidemia activity and could significantly inhibit lipid accumulation and TG levels in the liver [10,12]. Recently, we also found that the chlorogenic acid derivatives (chlorogenic acid diketal, 2, Figure 1) could still significantly decrease lipid accumulation in oleic acid-elicited HepG2 cells, in spite of the ketal substituent on the 1,4,5-position hydroxyl and carboxyl group. However, despite the promising and diverse pharmacological properties of caffeoylquinic acid analogues, synthesis and investigation of the structure–activity relationships of caffeoylquinic acid derivatives’ lipid-decreasing effect has not been discussed yet. 

**Figure 2:**
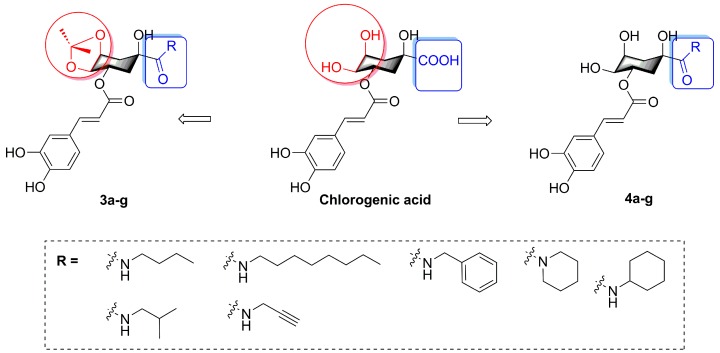
The structures of chlorogenic acid derivatives.

In this present research, we describe the synthesis of fourteen novel caffeoylquinic acid derivatives with various amines (n-butylamine, isobutylamine, n-octylamine, propargylamine, benzylamine, piperidine, and cyclohexylamine) which were appended to the 1-position carboxyl group of chlorogenic acid. Meanwhile, in view of the remaining chlorogenic acid diketal attenuating lipid accumulation activity, we also designed the amide derivatives with 4, 5-position hydroxyl groups substituted by ketal (isopropylidene). Therefore, the purpose of this study was to synthesize novel caffeoylquinic acid derivatives (**3a**–**g**, **4a**–**g**, Figure 2) and investigate the effects of derivatives on oleic acid-elicited lipid accumulation in HepG2 cells. The underlying lipid-lowering effect mechanisms of derivatives were also elucidated using intracellular TG quantification.

## 2. Results and Discussion

### 2.1. Chemistry

**Scheme 1:**
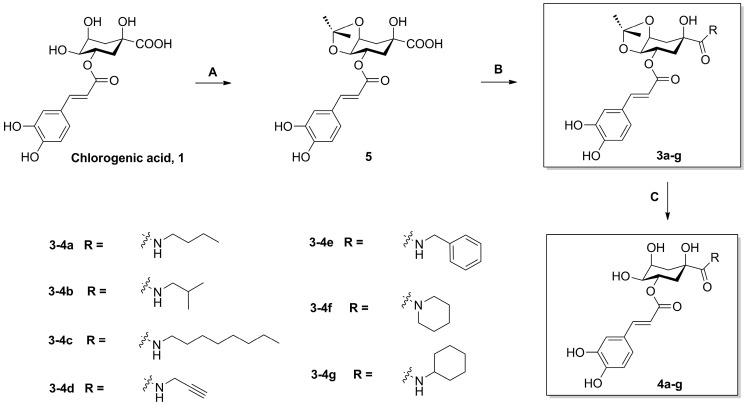
The synthesis of compounds **3a**–**g** and **4a**–**g**. Reagents and conditions: (**A**) DMP, TsOH, acetone; (**B**) BOP, DIEA, amines; (**c**) TFA, DCM, H_2_O (9:1:1).

The synthesis of derivatives **3a**–**g** and **4a**–**g** is outlined in Scheme 1. The naturally abundant chlorogenic acid (**1**) was treated with dimethyoxypropane (DMP) and p-toluenesulfonic acid (TsOH) in dry acetone to obtain chlorogenic acid ketal derivative **5**. Compounds **3a**–**g** were attained via amidation with various amines (n-butylamine, isobutylamine, n-octylamine, propargylamine, benzylamine, piperidine, and cyclohexylamine) of 1-position carboxyl group of compound **5** in BOP (benzotriazol-1-yl-oxytris (dimethylamino) phosphoniumhexa-fluorophosphate) and DIEA (dimethyltriethylamine) conditions, and then followed by deprotection at 4,5-position ketal in the presence of a TFA/DCM (trifluoroacetic acid/ dichloromethane) solution, to gain compounds **4a**–**g**. 

### 2.2. Biological Results and Discussion

#### 2.2.1. Lipid-Lowering Effects of Chlorogenic Acid and Compounds **3a**–**g** and **4a**–**g** on Oleic Acid-Elicited Lipid Accumulation in HepG2 Liver Cells 

**Figure 3:**
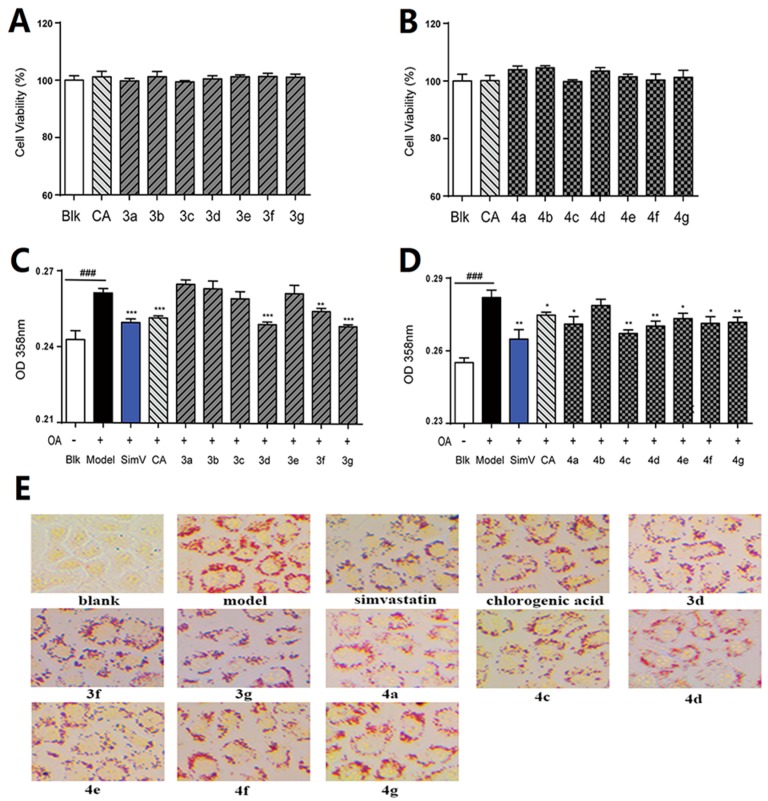
The nontoxic effects of chlorogenic acid and its derivatives on cell viability. (**A**) Cells treated with 10 μmol/L compounds **3a**–**3g**, for 24 h, respectively, and (**B**) compounds **4a**–**g,** for 24 h, respectively. The regulation effects of chlorogenic acid and its derivatives on oleic acid-elicited lipid accumulation in HepG2 liver cells. HepG2 cells were co-incubated in serum-free DMEM containing 100 μmol/L oleic acid (OA) (Model) and co-treated with 10 μmol/L of simvastatin (SimV) or chlorogenic acid (CA), (**C**) compounds **3a**–**g**, for 24 h, respectively, and (**D**) compounds **4a**–**g**, for 24 h, respectively. (**E**) Oil red O staining images. The data are expressed as the mean ± SEM (*n* = 8) from three independent experiments. ### *p* < 0.001 versus blank group (Blk); * *p* < 0.05 versus OA-treated cells; ** *p* < 0.01 versus OA-treated cells; *** *p* < 0.001 versus OA-treated cells.

**Figure 4:**
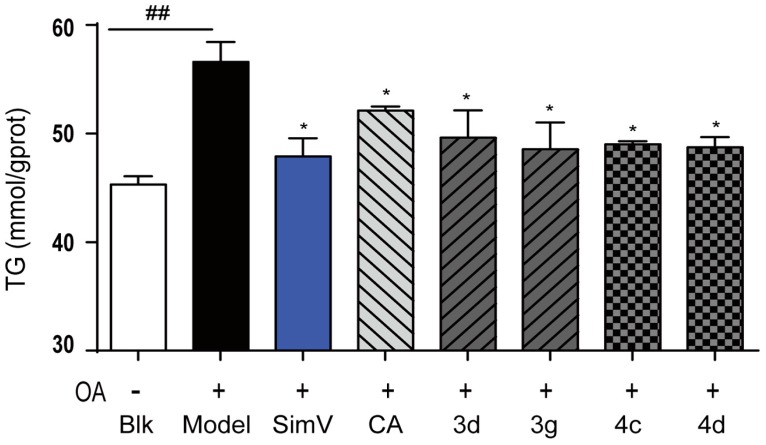
Inhibitory effects of compounds toward triglycerides in HepG2 liver cells. Positive control: simvastatin (SimV, 47.89%) and chlorogenic acid (CA, 51.51%); blank (Blk, 45.30%): DMEM; OA (56.61%): oleic acid. Intracellular levels of triglycerides were measured by kits according to the manufacturer’s instructions. Bars depict the means ± SEM in triplicate. ## *p* < 0.01 versus blank group (Blk); * *p* < 0.05 versus OA-treated cells.

The lipid-lowering effects of chlorogenic acid and its derivatives against oleic acid-elicited lipid accumulation in HepG2 liver cells were detected using the Oil red O staining assay. The MTS assay indicated that 10 μmol/L chlorogenic acid derivatives **3a**–**3g** (Figure 3a) and **4a**–**4g** (Figure 3b) displayed no cytotoxicity, within 24 h, to HepG2 liver cells. Oil red O staining results showed that some derivatives exhibited moderate to good regulation effects on oleic acid-elicited lipid accumulation in HepG2 liver cells. As shown in Figure 3c–d, the preliminary test of compounds **3a**–**g** and **4a**–**g** at 10 μmol/L revealed that **3d**, **3f**, **3g**, **4a**, and **4c**–**g** better moderated the lipid-lowering effects of HepG2 cells. Treatment of HepG2 liver cells with compounds **3d**, **3g**, **4a**, and **4c**–**g** exhibited better regulating effects than chlorogenic acid (CA). The absorbance decreased from 0.261 (with oleic acid treatment alone) to 0.249, 0.255, and 0.249 after treatment with 10 μmol/L compounds **3d**, **3f**, and **3g** in Figure 3c, respectively. In Figure 3d, the absorbance decreased from 0.282 (oleic acid alone) to 0.271, 0.267, 0.270, 0.273, 0.271, and 0.272 after treatment with 10 μmol/L derivatives **4a** and **4c**–**g** in Figure 3b, respectively. The Oil red O staining images are shown in Figure 3e. Among them, derivatives **3d**, **3g**, **4c**, and **4d** exhibited more potent regulation effects than the others, after treatment for 24 h, which suggests that **3d**, **3g**, **4c**, and **4d** deserve further evaluation as potential hypolipidemic agents for regulation on oleic acid-elicited lipid accumulation in HepG2 liver cells. 

The primary structure-activity relationships (SARs) suggested that, compared to the positive groups, simvastatin (SimV) and chlorogenic acid (CA), the amide derivatives of chlorogenic acid with 4, 5-position hydroxyl groups substituted by ketal had weaker potency than those with 4, 5-position hydroxyl groups exposed. Beyond that, the derivatives **3**–**4d**, **3**–**4f**, and **3**–**4g** with propynylamine, piperidine and cyclohexylamine groups could better regulate lipid accumulation after oleic acid treatment, indicating that the introduction of propargyl, piperidyl, and cyclohexyl groups could ameliorate lipid accumulation. Among them, derivative **3**–**4d** exhibited more potent lipid-lowering effects compared to the other compounds, which means that the propargyl group is a favourable substituent for regulation of lipid accumulation in HepG2 liver cells. However, derivatives **3**–**4b**, including isobutyl group, were inert, suggesting that the isobutyl group was negative factor in the derivatives. In addition, the above derivatives show lipid-lowering effects in Figure 3a–b, illustrating that the 1-position carboxyl group and 4, 5-position hydroxyl groups of chlorogenic acid were not essential groups for regulating lipid effects and different groups on the 1-position carboxyl group influenced lipid-lowering activities obviously. 

#### 2.2.2. Inhibitory Effects of Compounds **3d**, **3g**, **4c**, and **4d** Toward Triglycerides

On account of the preferable potency and typical structure, derivatives **3d**, **3g**, **4c**, and **4d** were chosen for further investigation with simvastatin and chlorogenic acid. We measured the levels of TG in HepG2 liver cells and found that consumptions of **3d**, **3g**, **4c**, and **4d** markedly reduced the content of TG in liver cells (49.63%, 48.56%, 49.00%, and 48.72% by 10 μmol/L derivatives, respectively). The efficacy of **3d**, **3g**, **4c**, and **4d** in decreasing TG levels was a little weaker than that of simvastatin, but better than that of chlorogenic acid, in decreasing TG levels at 10 μmol/L (Figure 4). Taken together, these results indicate that derivatives **3d**, **3g**, **4c**, and **4d** inhibited TG levels markedly on oleic acid-elicited lipid accumulation in HepG2 liver cells.

## 3. Experimental Section

### 3.1. General Information

All the reagents were used without further purification unless otherwise specified. Solvents were dried and redistilled prior to use in the usual manner. Analytical TLC was performed using silica gel HF254. Preparative column chromatography was performed with silica gel H. ^1^H and ^13^C-NMR spectra were recorded on a Bruker Advance III 600 MHz spectrometer. HRMS was obtained on a Thermofisher LTQ-Obitrap XL. Chlorogenic acid, DMP (Dimethyoxypropane), TsOH (p-Toluenesulfonic acid), BOP (Benzotriazol-1-yl-oxytris (dimethylamino)phosphoniumhexa-fluorophosphate), DIEA (Dimethyltriethylamine), TFA (trifluoroacetic acid), and DCM (dichloromethane) were purchased from the Energy Chemical Company. Dulbecco’s modified Eagle medium (DMEM) fetal bovine serum was purchased from Corning Inc (Corning, CA, USA). Penicillin and streptomycin were procured from Hyclone (South Logan, UT, USA). Simvastatin, oil-red O, oleic acid (OA), and dimethyl Sulphoxide DMSO were purchased from Sigma-Aldrich (St. Louis, MO, USA). The kits for Triglyceride (TG) were purchased from Jian Cheng Biotechnology Company (Nanjing, China). The Total RNA extraction reagent Trizol (Ambion, Austin, TX, USA), the PrimeScript RT reagent kit, and the SYBR-Green PCR kit were purchased from Transgene Biotech, Inc. (Beijing, China).

### 3.2. Chemistry

#### 3.2.1. Procedure for the Synthesis of Intermediate **5**

To a suspension of chlorogenic acid (7.0 g, 19.8 mmol) in dry acetone (60 mL) and DMP (40 mL), catalytic amount of TsOH (50 mg, 0.26 mmol) was added. Then the reaction mixture was stirred at room temperature for 30 min. Reaction was monitored by TLC. The crude mixture was neutralized with Na_2_CO_3_ powder to pH 6. Then the suspension was filtered out and the filtrate was evaporated and the crude product was subjected to column chromatography (eluent: PE-EtOAc, 1:1) to offer a pure light-yellow solid compound **5** (6.8 g, 87% yield).

#### 3.2.2. General Procedure for the Synthesis of Compounds **3a**–**g**

To a mixture of compound 5 (2.4 g, 6.1 mmol) and BOP (2.7 g, 6.1 mmol) in dry THF (100 mL), organic base DIEA (1.6 g, 12.2 mmol) was added and stirred at room temperature under N_2_ air. Then various amines (n-butylamine, isobutylamine, n-octylamine, propargylamine, benzylamine, piperidine, and cyclohexylamine) (6.7 mmol) were dropped and reacted respectively for 4**–**16 h. When complete, the reaction solvent was evaporated and the crude product was subjected to column chromatography (eluent: DCM-CH_3_OH, 10:1) to gain pure compound **3a**–**g** as a light-yellow solid.

*1-N-n-butyl-4, 5-di-O-Isopropylidene-Chlorogenic Acid Amide* (**3a**): Yellowish powder, 81% yield; 1H-NMR (600 MHz, DMSO) *δ*: 9.58(s, 1H, Ph-OH), 9.14(s, 1H, Ph-OH), 7.71 (t, *J* = 6.0 Hz, 1H, NH), 7.48 (d, *J* = 15.8 Hz, 1H, H-7′), 7.05 (d, *J* = 1.7 Hz, 1H, H-2′), 7.00 (dd, *J* = 8.3 Hz, 1.7 Hz, 1H, H-6′), 6.77 (d, *J* = 8.2 Hz, 1H, H-5′), 6.24 (d, *J* = 15.9 Hz, 1H, H-8′), 5.41 (s, 1H, H-1), 5.37–5.32 (m, 1H, H-3), 4.43–4.41 (m, 1H, H-4), 4.13–4.11 (m, 1H, H-5), 3.11–3.01 (m, 2H, NH-CH_2_), 2.26–2.22 (m, 1H, H-2), 1.99–1.94 (m, 1H, H-2), 1.80–1.73 (m, 2H, H-6), 1.42 (s, 3H, CH_3_-C-O), 1.39–1.36 (m, 2H, NH-CH_2_-CH_2_), 1.26–1.22 (m, 5H, CH_3_-C-O, CH_2_-CH_3_), 0.84 (t, *J* =7.3 Hz, 3H, CH_2_-CH_3_); ^13^C-NMR (150 MHz, DMSO) *δ*: 175.0, 165.9, 148.4, 145.6, 145.3, 125.6, 121.4, 115.8, 114.9, 114.1, 108.0, 76.9, 74.1, 73.5, 70.9, 38.2, 37.0, 34.5, 31.2, 28.0, 25.9, 19.4, 13.7; HRMS (ESI): Calcd for [M + Na]^+^ C_23_H_31_NNaO_8_: 472.1947, found 472.1948.

*1-N-isobutyl-4,5-di-O-Isopropylidene-ChlorogenicAcidAmide* (**3b**): Yellowish powder, 77% yield; ^1^H-NMR (600 MHz, DMSO) *δ*: 7.70 (t, *J* = 6.1 Hz, 1H, NH), 7.47 (d, *J* = 15.8 Hz, 1H, H-7′), 7.04 (d, *J* = 2.0 Hz, 1H, H-2′), 7.00 (dd, *J* = 8.2 Hz, 2.0 Hz, 1H, H-6′), 6.76 (d, *J* = 8.2 Hz, 1H, H-5′), 6.24 (d, *J* = 15.9 Hz, 1H, H-8′), 5.47 (br, 1H, H-1), 5.37–5.33 (m, 1H, H-3), 4.43–4.41 (m, 1H, H-4), 4.14–4.11 (m, 1H, H-5), 2.94–2.86 (m, 2H, NH-CH_2_), 2.26–2.22 (m, 1H, H-2), 1.99–1.96 (m, 1H, H-2), 1.80–1.75 (m, 2H, H-6), 1.74–1.69 (m, 1H, NH-CH_2_-CH), 1.42 (s, 3H, CH_3_-C-O), 1.26 (s, 3H, CH_3_-C-O), 0.82–0.80 (m, 6H, 2×CH_3_); ^13^C-NMR (150 MHz, DMSO) *δ*: 175.1, 165.9, 148.5, 145.6, 145.4, 125.6, 121.4, 115.8, 114.9, 114.1, 108.0, 76.9, 74.2, 73.6, 70.9, 45.9, 37.1, 34.4, 28.1, 26.0, 20.0; HRMS (ESI): Calcd for [M + Na]^+^ C_23_H_31_NNaO_8_: 472.1947, found 472.1946.

*1-N-n-octyl-4,5-di-O-Isopropylidene-ChlorogenicAcidAmide* (**3c**): Yellowish powder, 82% yield; ^1^H-NMR (600 MHz, DMSO) *δ*: 7.74 (t, *J* = 5.9 Hz, 1H, NH), 7.47 (d, *J* = 15.8 Hz, 1H, H-7′), 7.04 (d, *J* = 2.0 Hz, 1H, H-2′), 7.00 (dd, *J* = 8.2 Hz, 2.0 Hz, 1H, H-6′), 6.76 (d, *J* = 8.2 Hz, 1H, H-5′), 6.24 (d, *J* = 15.9 Hz, 1H, H-8′), 5.44 (br, 1H, H-1), 5.36–5.31 (m, 1H, H-3), 4.43–4.40 (m, 1H, H-4), 4.13–4.11 (m, 1H, H-5), 3.10–2.98 (m, 2H, NH-CH_2_), 2.25–2.22 (m, 1H, H-2), 1.97–1.94 (m, 1H, H-2), 1.75–1.74 (m, 2H, H-6), 1.41 (s, 3H, CH_3_-C-O), 1.40–1.37 (m, 2H, NH-CH_2_-CH_2_), 1.25 (s, 3H, CH_3_-C-O), 1.25–1.19 (m, 10H, (CH_2_)_5_-CH_3_), 0.83 (t, *J* =7.1 Hz, 3H, (CH_2_)_5_-CH_3_); ^13^C-NMR (150 MHz, DMSO) *δ*: 174.9, 165.8, 148.4, 145.5, 145.2, 125.5, 121.3, 115.7, 114.8, 114.0, 107.9, 76.8, 73.9, 73.4, 70.8, 38.4, 37.0, 34.5, 31.1, 28.9, 28.6, 28.5, 27.9, 26.1, 25.8, 22.0, 13.8; HRMS (ESI): Calcd for [M + Na]^+^ C_27_H_39_NNaO_8_: 528.2573, found 528.2578.

*1-N-propargyl-4,5-di-O-Isopropylidene-ChlorogenicAcidAmide* (**3d**): Yellowish powder, 75% yield; ^1^H-NMR (600 MHz, DMSO) *δ*: 8.16 (t, *J* = 5.8 Hz, 1H, NH), 7.47 (d, *J* = 15.8 Hz, 1H, H-7′), 7.04 (d, *J* = 2.0 Hz, 1H, H-2′), 7.00 (dd, *J* = 8.2 Hz, 2.0 Hz, 1H, H-6′), 6.76 (d, *J* = 8.2 Hz, 1H, H-5′), 6.24 (d, *J* = 15.8 Hz, 1H, H-8′), 5.50 (s, 1H, H-1), 5.35–5.31 (m, 1H, H-3), 4.43–4.41 (m, 1H, H-4), 4.14–4.12 (m, 1H, H-5), 3.84 (dd, *J* = 5.8 Hz, 2.5 Hz, 2H, NH-CH_2_), 3.03 (t, *J* = 2.5 Hz, 1H, CCH), 2.25–2.22 (m, 1H, H-2), 2.00–1.95 (m, 1H, H-2), 1.79–1.70 (m, 2H, H-6), 1.42 (s, 3H, CH_3_-C-O), 1.26 (s, 3H, CH_3_-C-O); ^13^C-NMR (150 MHz, DMSO) *δ*: 175.3, 166.0, 148.5, 145.7, 145.5, 125.7, 121.5, 115.9, 114.9, 114.2, 108.2, 81.2, 76.9, 74.2, 73.6, 72.6, 70.9, 37.0, 34.3, 28.4, 28.1, 26.0; HRMS (ESI): Calcd for [M + Na]^+^ C_22_H_25_NNaO_8_: 454.1478, found 454.1480.

*1-N-benzyl-4, 5-di-O-Isopropylidene-Chlorogenic Acid Amide* (**3e**): Yellowish powder, 79% yield; ^1^H-NMR (600 MHz, DMSO) *δ*: 8.32 (t, *J* = 6.2 Hz, 1H, NH), 7.48 (d, *J* = 15.8 Hz, 1H, H-7′), 7.31–7.28 (m, 2H, Ph-H), 7.24–7.20 (m, 3H, Ph-H), 7.05 (d, *J* = 2.0 Hz, 1H, H-2′), 7.01 (dd, *J* = 8.2 Hz, 2.0 Hz, 1H, H-6′), 6.77 (d, *J* = 8.2 Hz, 1H, H-5′), 6.25 (d, *J* = 15.9 Hz, 1H, H-8′), 5.55 (br, 1H, H-1), 5.39–5.35 (m, 1H, H-3), 4.45–4.42 (m, 1H, H-4), 4.32–4.25 (m, 2H, NH-CH_2_), 4.15–4.13 (m, 1H, H-5), 2.29–2.25 (m, 1H, H-2), 2.04–2.01 (m, 1H, H-2), 1.84–1.76 (m, 2H, H-6), 1.43 (s, 3H, CH_3_-C-O), 1.26 (s, 3H, CH_3_-C-O); ^13^C-NMR (150 MHz, DMSO) *δ*: 175.2, 165.8, 148.4, 145.5, 145.3, 139.5, 128.1, 126.9, 126.7, 125.5, 121.3, 115.7, 114.8, 114.0, 108.0, 76.8, 74.1, 73.4, 70.8, 42.0, 37.0, 34.5, 27.9, 25.8; HRMS (ESI): Calcd for [M + Na]^+^ C_26_H_29_NNaO_8_: 506.1791, found 506.1790.

*1-N-piperidyl-4, 5-di-O-Isopropylidene-Chlorogenic Acid Amide* (**3f**): Yellowish powder, 64% yield; ^1^H-NMR (600 MHz, DMSO) *δ*: 9.59(s, 1H, Ph-OH), 9.15(s, 1H, Ph-OH), 7.48 (d, *J* = 15.8 Hz, 1H, H-7′), 7.04 (d, *J* = 1.5 Hz, 1H, H-2′), 7.00 (dd, *J* = 8.1 Hz, 1.5 Hz, 1H, H-6′), 6.77 (d, *J* = 8.0 Hz, 1H, H-5′), 6.22 (d, *J* = 15.9 Hz, 1H, H-8′), 5.62 (s, 1H, H-1), 5.32–5.29 (m, 1H, H-3), 4.45–4.42 (m, 1H, H-4), 4.08–4.06 (m, 1H, H-5), 3.97–3.59 (m, 2H, NH-CH_2_), 3.55–3.43 (m, 1H, NH-CH), 3.35–3.17 (m, 1H, NH-CH), 2.33–2.30 (m, 1H, H-2), 2.01–1.98 (m, 1H, H-2), 1.90–1.86 (m, 2H, H-6), 1.58–1.50 (m, 2H, CH_2_), 1.49–1.37 (m, 2H, CH_3_-C-O, (CH_2_)_2_), 1.25 (m, 3H, CH_3_-C-O); ^13^C-NMR (150 MHz, DMSO) *δ*: 171.5, 165.8, 148.5, 145.6, 145.4, 125.5, 121.3, 115.8, 114.8, 114.0, 108.0, 76.1, 74.8, 73.2, 70.6, 37.0, 36.8, 34.5, 29.0, 27.9, 25.8, 25.5, 24.2; HRMS (ESI): Calcd for [M + Na]^+^ C_24_H_31_NNaO_8_: 484.1947, found 484.1949.

*1-N-cyclohexyl-4, 5-di-O-Isopropylidene-Chlorogenic Acid Amide* (**3g**): Yellowish powder, 71% yield; ^1^H-NMR (400 MHz, DMSO) *δ*: 7.47 (d, *J* = 15.8 Hz, 1H, H-7′), 7.38 (d, *J* = 8.2 Hz, 1H, NH), 7.04–6.99 (m, 2H, H-2′, 6′), 6.76 (d, *J* = 8.1 Hz, 1H, H-5′), 6.24 (d, *J* = 15.8 Hz, 1H, H-8′), 5.44 (br, 1H, H-1), 5.37–5.30 (m, 1H, H-3), 4.42–4.41 (m, 1H, H-4), 4.14–4.11 (m, 1H, H-5), 3.51–3.49 (m, 1H, NH-CH), 2.25–2.20 (m, 1H, H-2), 1.97–1.91 (m, 1H, H-2), 1.77–1.75(m, 2H, H-6), 1.67–1.65 (m, 4H, CH(CH_2_)_2_), 1.41 (s, 3H, CH_3_-C-O), 1.26–1.10 (m, 9H, CH_3_-C-O, (CH_2_)_3_); ^13^C-NMR (100 MHz, DMSO) *δ*: 174.1, 165.8, 148.4, 145.5, 145.3, 125.5, 121.3, 115.7, 114.8, 114.0, 108.0, 76.8, 74.0, 73.5, 70.9, 47.4, 37.0, 34.4, 32.1, 28.0, 25.9, 25.1, 24.4; HRMS (ESI): Calcd for [M + Na]^+^ C_25_H_33_NNaO_8_: 498.2104, found 498.2106.

#### 3.2.3. General Procedure for the Synthesis of Compounds **4a**–**g**

A mixture of compound **3a**–**g** (1.7 mmol) and TFA-DCM-H_2_O (10 mL) was stirred and reacted for 4 h at room temperature. Then the reaction solvent was concentrated and the residue was purified by silica gel column chromatography (eluent: DCM-CH_3_OH, 8:1) to get pure compound 4a-g as a light-yellow solid.

*1-N-n-butyl-ChlorogenicAcidAmide* (**4a**): Yellowish powder, 78% yield; ^1^H-NMR (600 MHz, MeOD) *δ*: 7.58 (d, *J* = 15.9 Hz, 1H, H-7′), 7.05 (d, *J* = 1.9 Hz, 1H, H-2′), 6.95 (dd, *J* = 8.2 Hz, 1.9 Hz, 1H, H-6′), 6.78 (d, *J* = 8.1 Hz, 1H, H-5′), 6.29 (d, *J* = 15.9 Hz, 1H, H-8′), 5.42–5.38 (m, 1H, H-3), 4.24–4.23 (m, 1H, H-4), 3.72–3.70 (m, 1H, H-5), 3.21–3.19 (m, 2H, NH-CH_2_), 2.12–2.07 (m, 2H, H-2), 2.02–2.00 (m, 1H, H-6), 1.95–1.91 (m, 1H, H-6), 1.52–1.47 (m, 2H, NH-CH_2_-CH_2_), 1.36–1.32 (m, 2H, CH_2_-CH_3_), 0.93 (t, *J* = 7.4 Hz, 3H, CH_2_-CH_3_); ^13^C-NMR (150 MHz, MeOD) *δ*: 176.6, 169.0, 149.6, 147.0, 146.8, 127.8, 122.9, 116.5, 115.4, 115.2, 77.8, 74.4, 72.7, 71.9, 40.0, 39.9, 38.7, 32.6, 21.0, 14.1; HRMS (ESI): Calcd for [M + Na]^+^ C_20_H_27_NNaO_8_: 432.1634, found 432.1636.

*1-N-Isobuty-Chlorogenic Acid Amide* (**4b**): Yellowish powder, 85% yield; ^1^H-NMR (600 MHz, MeOD) *δ*: 7.57 (d, *J* =15.7 Hz, 1H, H-7′), 7.05 (s, 1H, H-2′), 6.99–6.89 (m, 1H, H-6′), 6.78 (d, *J* = 7.7 Hz, 1H, H-5′), 6.29 (d, *J* =15.7 Hz, 1H, H-8′), 5.46–5.37 (m, 1H, H-3), 4.30–4.18 (m, 1H, H-4), 3.78–3.66 (m, 1H, H-5), 3.09–2.95 (m, 2H, NH-CH_2_), 2.11–1.94 (m, 1H, H-2, 6), 1.85–1.73 (m, 1H, NH-CH_2_-CH), 0.94–0.84 (m, 6H, 2 × CH_3_); ^13^C-NMR (150 MHz, MeOD) *δ*: 176.7, 169.0, 149.5, 147.0, 146.7, 127.7, 122.9, 116.5, 115.3, 115.2, 77.8, 74.3, 72.6, 71.9, 47.6, 40.0, 38.7, 29.6, 20.3; HRMS (ESI): Calcd for [M + Na]^+^ C_20_H_27_NNaO_8_: 432.1634, found 432.1638.

*1-N-n-octyl-ChlorogenicAcidAmide* (**4c**): Yellowish powder, 76% yield; ^1^H-NMR (400 MHz, MeOD) *δ*: 7.58 (d, *J* = 15.9 Hz, 1H, H-7′), 7.05 (d, *J* = 1.9 Hz, 1H, H-2′), 6.95 (dd, *J* = 8.2 Hz, 1.9 Hz, 1H, H-6′), 6.78 (d, *J* = 8.2 Hz, 1H, H-5′), 6.29 (d, *J* = 15.9 Hz, 1H, H-8′), 5.43–5.37 (m, 1H, H-3), 4.24–4.23 (m, 1H, H-4), 3.72–3.69 (m, 1H, H-5), 3.20–3.17 (m, 2H, NH-CH_2_), 2.13–1.90 (m, 1H, H-2, 6), 1.52–1.49 (m, 2H, NH-CH_2_-CH_2_), 1.34–1.26 (m, 10H, (CH_2_)_5_-CH_3_), 0.87 (t, *J* =7.1 Hz, 3H, -CH_3_); ^13^C-NMR (100 MHz, MeOD) *δ*: 176.6, 169.0, 149.6, 147.0, 146.8, 127.8, 123.0, 116.5, 115.3, 115.1, 77.7, 74.4, 72.7, 72.0, 40.2, 40.0, 38.7, 33.0, 30.5, 30.4, 27.9, 23.7, 14.4; HRMS (ESI): Calcd for [M + Na]^+^ C_24_H_35_NNaO_8_: 488.2260, found 488.2252.

*1-N-Propargyl-Chlorogenic Acid Amide* (**4d**): Yellowish powder, 69% yield; ^1^H-NMR (400 MHz, MeOD) *δ*: 7.57 (d, *J* = 15.9 Hz, 1H, H-7′), 7.11–7.00 (m, 1H, H-2′), 6.99–6.89 (m, 1H, H-6′), 6.78 (d, *J* = 8.0 Hz, 1H, H-5′), 6.29 (d, *J* = 15.9 Hz, 1H, H-8′), 5.43–5.37 (m, 1H, H-3), 4.29–4.19 (m, 1H, H-4), 4.03–4.92 (m, 2H, NH-CH_2_), 3.74–3.71 (m, 1H, H-5), 2.61–2.49 (m, 1H, CCH), 2.15–1.95 (m, 4H, H-2, 6); ^13^C-NMR (100 MHz, MeOD) *δ*: 176.5, 169.0, 149.5, 147.0, 146.7, 127.7, 123.0, 116.5, 115.3, 115.1, 80.5, 77.8, 74.3, 72.5, 72.1, 71.8, 39.8, 38.5, 29.4; HRMS (ESI): Calcd for [M + Na]^+^ C_19_H_21_NNaO_8_: 414.1165, found 414.1667.

*1-N-benzyl-Chlorogenic Acid Amide* (**4e**): Yellowish powder, 73% yield; ^1^H-NMR (600 MHz, MeOD) *δ*: 7.58 (d, *J* = 15.9 Hz, 1H, H-7′), 7.32–7.22 (m, 5H, Ph-H), 7.05 (s, 1H, H-2′), 6.96–6.94 (m, 1H, H-6′), 6.78 (d, *J* = 8.1 Hz, 1H, H-5′), 6.29 (d, *J* = 15.8 Hz, 1H, H-8′), 5.45–5.39 (m, 1H, H-3), 4.39 (m, 2H, NH-CH_2_), 4.26–4.25 (m, 1H, H-4), 3.73–3.69 (m, 1H, H-5), 2.17–1.97 (m, 4H, H-2, 6); ^13^C-NMR (150 MHz, MeOD) *δ*: 176.8, 169.0, 149.5, 147.0, 146.8, 139.9, 129.5, 128.2, 128.2, 128.0, 122.9, 116.5, 115.4, 115.2, 77.9, 74.4, 72.7, 71.9, 43.8, 40.0, 38.8; HRMS (ESI): Calcd for [M + Na]^+^ C_23_H_25_NNaO_8_: 466.1448, found 466.1484.

*1-N-piperidyl-ChlorogenicAcidAmide* (**4f**): Yellowish powder, 61% yield; ^1^H-NMR (600 MHz, MeOD) *δ*: 7.57 (d, *J* = 15.9 Hz, 1H, H-7′), 7.05 (d, *J* = 1.9 Hz, 1H, H-2′), 6.95 (dd, *J* = 8.2 Hz, 2.0 Hz, 1H, H-6′), 6.78 (d, *J* = 8.2 Hz, 1H, H-5′), 6.26 (d, *J* = 15.9 Hz, 1H, H-8′), 5.36–5.33 (m, 1H, H-3), 4.28–4.27 (m, 1H, H-4), 4.06–3.79 (m, 2H, NH-CH_2_), 3.73–3.71 (m, 1H, H-5), 3.63–3.38 (m, 2H, NH-CH_2_), 2.33–2.01 (m, 4H, H-2, 6), 1.68–1.61 (m, 2H, CH_2_), 1.60–1.48 (m, 4H, (CH_2_)_2_), 1.90–1.86 (m, 2H, H-6), 1.58–1.50 (m, 2H, CH_2_), 1.49–1.37 (m, 2H, CH_3_-C-O, (CH_2_)_2_); ^13^C-NMR (150 MHz, MeOD) *δ*: 173.2, 168.8, 149.6, 147.2, 146.8, 127.8, 123.0, 116.5, 115.3, 115.2, 78.6, 73.3, 72.4, 71.6, 40.0, 39.2, 38.7, 30.7, 25.6; HRMS (ESI): Calcd for [M + Na]^+^ C_21_H_27_NNaO_8_: 444.1634, found 444.1637.

*1-N-cyclohexyl-chlorogenicacidamide* (**4g**): Yellowish powder, 64% yield; ^1^H-NMR (600 MHz, MeOD) *δ*: 7.57 (d, *J* = 15.9 Hz, 1H, H-7′), 7.05 (d, *J* = 2.0 Hz, 1H, H-2′), 6.95 (dd, *J* = 8.2 Hz, 2.0 Hz, 1H, H-6′), 6.78 (d, *J* = 8.2 Hz, 1H, H-5′), 6.29 (d, *J* =16.0 Hz, 1H, H-8′), 5.42–5.36 (m, 1H, H-3), 4.24–4.22 (m, 1H, H-4), 3.72–3.69 (m, 1H, H-5), 3.65–3.58 (m, 1H, NH-CH), 2.12–1.89 (m, 4H, H-2, 6), 1.84–1.82 (m, 2H, CH_2_), 1.77–1.73(m, 2H, CH_2_), 1.65–1.58 (m, 2H, CH_2_), 1.40–1.34 (m, 2H, CH_2_), 1.26–1.23 (m, 2H, CH_2_); ^13^C-NMR (150 MHz, MeOD) *δ*: 175.7, 169.0, 149.6, 147.0, 146.8, 127.8, 122.9, 116.5, 115.4, 115.2, 77.6, 74.4, 72.7, 72.0, 49.6, 40.0, 38.7, 33.6, 30.7, 26.5, 26.1; HRMS (ESI): Calcd for [M + Na]^+^ C_22_H_29_NNaO_8_: 458.1791, found 458.1795.

The spectrograms of the compounds **3a**–**g** and **4a**–**g** are shown in the Electronic Supplementary Material (ESM).

### 3.3. Evaluation of the Biological Activity

#### 3.3.1. Cell Culture

HepG2 cells were originated from American Type Culture Collection (ATCC) (Manassas, VA, USA) and obtained from the Peking Union Medical College (Beijing, China). HepG2 cells were cultured in Dulbecco’s modified Eagle medium (DMEM) supplemented with 10% fetal bovine serum, 1% penicillin, and streptomycin at 37 °C in a 5% CO_2_ atmosphere. When grown to 70%–80% confluence, cells were incubated in serum-free DMEM containing 100 μmol/L oleic acid and co-treated with 10 μmol/L of simvastatin or chlorogenic acid, compound **3a**–**g**, and **4a**–**g** for 24 h respectively. Cells maintained in serum-free DMEM were used as the blank control. Compounds were dissolved in dimethyl sulphoxide (DMSO) and an equal volume of it was added in the control group. 

#### 3.3.2. Cell Viability Assay

Cell viability was examined using MTS assay. HepG2 cells in 96-well culture plates were treated with 10 μmol/L compounds. The cells were incubated for 24 h and the MTS reagent was added to each well according to the instruction of CellTiter 96® Aqueous One Solution Cell Proliferation Assay (promega corperation, Beijing, China). The absorbance at 490 nm was measured using a microplate reader (ThermoFisher Ltd., Shanghai, China).

#### 3.3.3. Oil red O staining

The cells with 70–80% confluence in 96 well plates were incubated in serum-free DMEM + OA (oleic acid) (100 μmol/L) and 10 μmol/L of chlorogenic acid, compound **3a**–**g**, and **4a**–**g**, respectively, or the positive control simvastatin (10 µmol/L), for 24 h. Cells were then fixed with 4% w/v paraformal deyde (30 min, room temperature) and stained with 0.5% filtered oil-red O solution (15 min, room temperature). The staining was evaluated by a Tecan Infinite M1000Pro Microplate Reader and spectrophotometry at 358 nm.

#### 3.3.4. Intracellular TG Quantification

HepG2 cells with 70–80% confluence in 6 well plates were incubated in serum-free DMEM + OA (100 μmol/L) and 10 μmol/L of chlorogenic acid, compound **3a**–**g**, and **4a**–**g**, respectively, or the positive control simvastatin (10 µmol/L) for 24 h. The cells were subjected to TG quantification as introduced by the protocol of Triglyceride Quantification Kit. Each experiment was repeated in triplicate, with duplicates each.

#### 3.3.5. Statistical Analysis

Data are expressed as mean ± SEM. One-way ANOVA was used to determine significant differences among groups, after which the modified Students t-test with the Bonferroni correction was used for comparison between individual groups. All statistical analyses were performed with SPSS 17.0 software (SPSS Inc., Chicago, IL, USA). The value *p* < 0.05 was considered statistically significant.

## 4. Conclusions

In summary, fourteen caffeoylquinic acid derivatives of chlorogenic acid were designed, synthesized, and evaluated for their lipid-lowering effects. Several of the derivatives showed potent lipid-lowering activity against oleic acid-elicited lipid accumulation in HepG2 liver cells. Particularly, derivatives **3d**, **3g**, **4c,** and **4d** exhibited more potential lipid-lowering effect than the positive simvastatin and chlorogenic acid. Preliminary SAR analysis has shown that the propargyl group of the amide derivatives had a good impact on the protective effect. Further research on mechanism of **3d**, **3g**, **4c,** and **4d** revealed that it was related to the regulation of TG levels. These above results suggest that derivatives **3d**, **3g**, **4c,** and **4d** may be a promising therapeutic candidate as potential hypolipidemic agents. Further studies to design the derivatives of chlorogenic acid coupled with naturally occurring kinds of amino acids, as well as to explore the mechanism of action of this novel class of compounds is, planned to start in our group in the near future [21,22,23].

## Supplementary Materials

The following are available online.

## References

[1] Barry V.W., Caputo J.L., Kang M. (2018). The joint association of fitness and fatness on cardiovascular disease mortality: A meta-analysis. Prog. Cardiovasc. Dis..

[2] Britton K.A., Massaro J.M., Murabito J.M., Kreger B.E., Hoffmann U., Fox C.S. (2013). Body fat distribution, incident cardiovascular disease, cancer, and all-cause mortality. J. Am. Coll. Cardiol..

[3] Koliaki C., Liatis S., Kokkinos A. (2018). Obesity and cardiovascular disease: Revisiting an old relationship. Metabolism.

[4] Bellan M., Menegatti M., Ferrari C., Carnevale Schianca G.P., Pirisi M. (2018). Ultrasound-assessed visceral fat and associations with glucose homeostasis and cardiovascular risk in clinical practice. Nutr. Metab. Cardiovas..

[5] Elagizi A., Kachur S., Lavie C.J., Carbone S., Pandey A., Ortega F.B., Milani R.V. (2018). An overview and update on obesity and the obesity paradox in cardiovascular diseases. Prog. Cardiovasc. Dis..

[6] Cholesterol Treatment Trialists’ (CTT) Collaboration (2010). Efficacy and safety of more intensive lowering of LDL cholesterol: A meta-analysis of data from 170,000 participants in 26 randomised trials. Lancet.

[7] Berry J.D., Dyer A., Cai X., Garside D.B., Ning H.Y., Thomas A., Greenland P., Horn L.V., Tracy R.P., Lloyd-Jones D.M. (2012). Lifetime risks of cardiovascular disease. N. Engl. J. Med..

[8] Jones A. (2013). Triglycerides and cardiovascular risk. Heart.

[9] Pilz S., Scharnagl H., Tiran B., Seelhorst U., Wellnitz B., Boehm B.O., Schaefer J.R., März W. (2006). Free fatty acids are independently associated with all-cause and cardiovascular mortality in subjects with coronary artery disease. J. Clin. Endocrinol. Metab..

[10] Zhang X.P., Wu C.M., Wu H.F., Sheng L.H., Su Y., Zhang X., Luan H., Sun G.B., Sun X.B., Tian Y. (2013). Anti-hyperlipidemic effects and potential mechanisms of action of the caffeoylquinic acid-rich *Pandanus tectorius* fruit extract in hamsters fed a high fat-diet. PLoS ONE.

[11] Andriani Y., Ramli N.M., Syamsumir D.F., Kassim M.N.I., Jaafar J., Aziz N.A., Marlina L., Musa N.S., Mohamad H. (2015). Phytochemical analysis, antioxidant, antibacterial and cytotoxicity properties of keys and cores part of *Pandanus tectorius* fruits. Arab. J. Chem..

[12] Wu C.M., Zhang X.P., Zhang X., Luan H., Sun G.B., Sun X.B., Wang X.L., Guo P., Xu D.D. (2014). The caffeoylquinic acid-rich *Pandanus tectorius* fruit extract increases insulin sensitivity and regulates hepatic glucose and lipid metabolism in diabetic db/db mice. J. Nutr. Biochem..

[13] Agunloye O.M., Oboh G., Ademiluyi A.O., Ademosun A.O., Akindahunsi A.A., Oyagbemi A.A., Omobowale T.O., Ajibade T.O., Adedapo A.A. (2019). Cardio-protective and antioxidant properties of caffeic acid and chlorogenic acid: Mechanistic role of angiotensin converting enzyme, cholinesterase and arginase activities in cyclosporine induced hypertensive rats. Biomed. Pharmacother..

[14] Zhao M.M., Wang H.Y., Yang B., Tao H. (2010). Identification of cyclodextrin inclusion complex of chlorogenic acid and its antimicrobial activity. Food Chem..

[15] Gao R.F., Yang H.D., Jing S.F., Liu B., Wei M., He P.F., Zhang N.S. (2018). Protective effect of chlorogenic acid on lipopolysaccharide-induced inflammatory response in dairy mammary epithelial cells. Microb. Pathog..

[16] Wang D.Y., Zhao X.M., Liu Y.L. (2017). Hypoglycemic and hypolipidemic effects of a polysaccharide from flower buds of *Lonicera japonica* in streptozotocin-induced diabetic rats. Int. J. Biol. Macromol..

[17] Bender O., Llorent-Martínez E.J., Zengin G., Mollica A., Ceylan R., Molina-García L., Córdova M.L.F., Atalay A. (2018). Integration of in vitro and in silico perspectives to explain chemical characterization, biological potential and anticancer effects of *Hypericum salsugineum*: A pharmacologically active source for functional drug formulations. PLoS ONE.

[18] Llorent-Martínez E.J., Zengin G., Córdova M.L.F., Bender O., Atalay A., Ceylan R., Mollica A., Mocan A., Uysal S., Guler G.O. (2017). Traditionally used *Lathyrus* species: Phytochemical composition, antioxidant activity, enzyme inhibitory properties, cytotoxic effects, and in silico studies of *L. czeczottianus* and *L. nissolia*. Front. Pharmacol..

[19] Jabeur I., Tobaldini F., Martins N., Barros L., Martins I., Calhelha R.C., Henriques M., Silva S., Achour L., Santos-Buelga C. (2016). Bioactive properties and functional constituents of *Hypericum*
*androsaemum* L.: A focus on the phenolic profile. Food Res. Int..

[20] Truong V.D., Mcfeeters R.F., Thompson R.T., Dean L.L., Shofran B. (2007). Phenolic acid content and composition in leaves and roots of common commercial sweetpotato (*Ipomea batatas* L.) cultivars in the United States. J. Food Sci..

[21] Stefanucci A., Macedonio G., Dvorácskó S., Tömböly C., Mollica A. (2018). Novel fubinaca/rimonabant hybrids as endocannabinoid system modulators. Amino Acids.

[22] Monti L., Stefanucci A., Pieretti S., Marzoli F., Fidanza L., Mollica A., Mirzaie S., Carradori S., Petrocellis L.D., Moriello A.S. (2016). Evaluation of the analgesic effect of 4-anilidopiperidine scaffold containing ureas and carbamates. J. Enzyme. Inhib. Med. Chem..

[23] Mollica A., Costante R., Akdemir A., Carradori S., Stefanucci A., Macedonio G., Ceruso M., Supuran C.T. (2015). Exploring new probenecid-based carbonic anhydrase inhibitors: Synthesis, biological evaluation and docking studies. Bioorg. Med. Chem..

