# Supplementary material for: Synthesis and In Vitro Evaluation of Caffeoylquinic Acid Derivatives as Potential Hypolipidemic Agents
Source: Molecules. 2019 Mar 9;24(5):964. doi: 10.3390/molecules24050964 (PMC6429172; doi:10.3390/molecules24050964)
Supplement: Supplementary file 1 [file molecules-24-00964-s001.pdf]

Synthesis and *in vitro* evaluation of caffeoylquinic acids derivatives as potential hypolipidemic agents

Yu Tian[1,†], Xiao-xue Cao[1,†], Hai Shang[1], Chong-ming Wu[1], Xi Zhang[2], Peng Guo[1,*], Xiao-po Zhang[3,*], and Xu-Dong Xu[1,*]

[1] Beijing Key Laboratory of Innovative Drug Discovery of Traditional Chinese Medicine (Natural Medicine) and Translational Medicine; Key Laboratory of Bioactive Substances and Resources Utilization of Chinese Herbal Medicine, Ministry of Education; Key Laboratory of Efficacy Evaluation of Chinese Medicine against Glycolipid Metabolic Disorders, State Administration of Traditional Chinese Medicine; Zhong guan cun Open Laboratory of the Research and Development of Natural Medicine and Health Products; Key Laboratory of new drug discovery based on Classic Chinese medicine prescription; Institute of Medicinal Plant Development, Chinese Academy of Medical Sciences & Peking Union Medical College, Beijing 100193, P. R. China; ytian@implad.ac.cn (Y.T.); snow2018cxx@163.com (X.-X.C.); hshang@implad.ac.cn (H.S.); cmwu@implad.ac.cn (C.-M.W.)

[2] Center of Research and Development on Life Sciences and Environment Sciences, Harbin University of Commerce, Harbin 150076, P. R. China; 18800467885@163.com (X.Z.)

[3] School of Pharmacy, Hainan Medical Unversity, Haikou 571199, P. R. China

† These authors contributed equally to this work.

* Correspondence: pguo@ implad.ac.cn (P. G.); z_xp1412@163.com (X.-P.Z.); xdxu@implad.ac.cn (X.-D.X.)

*Correspondence authors. Address: Institute of Medicinal Plant Development, Chinese Academy of Medical Sciences & Peking Union Medical College, No. 151, Malianwa North Road, Haidian District, Beijing 100193, PR China. Tel: +86-010-57833296; Fax: +86-010-57833296.

E-mail addresses: xdxu@implad.ac.cn (Xudong Xu), pguo@implad.ac.cn (Guo Peng).

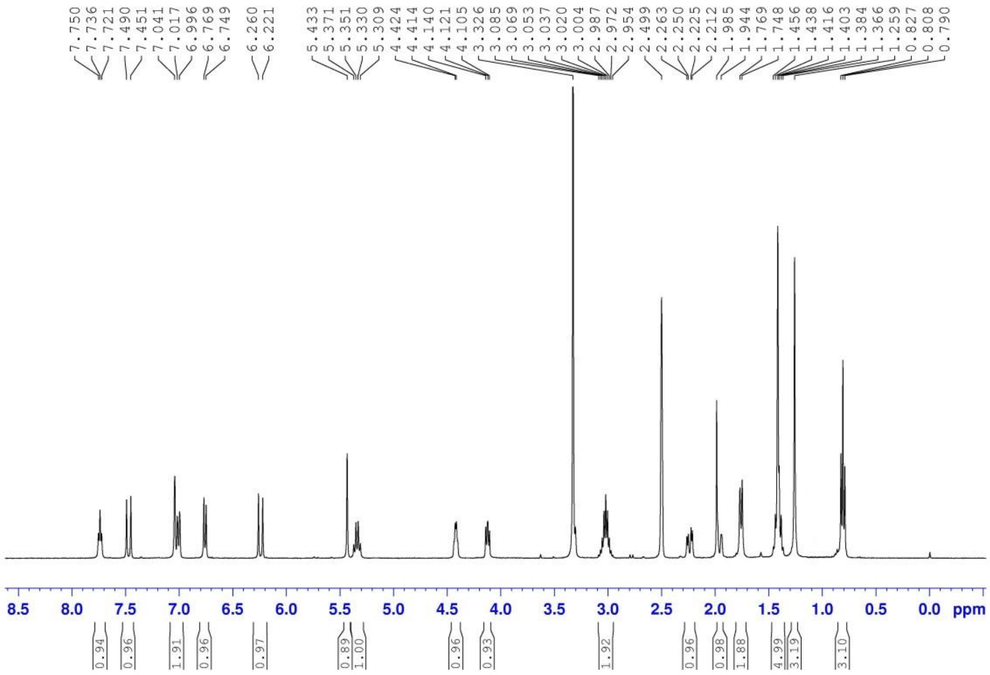

^{1}H NMR of compound **3a**

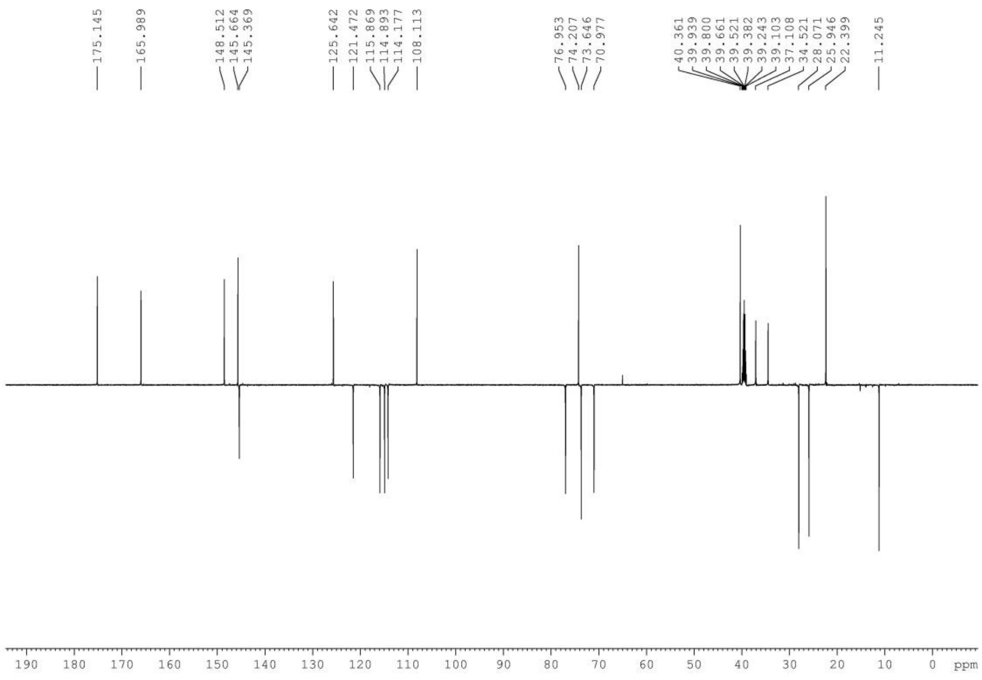

^{13}C NMR of compound **3a**

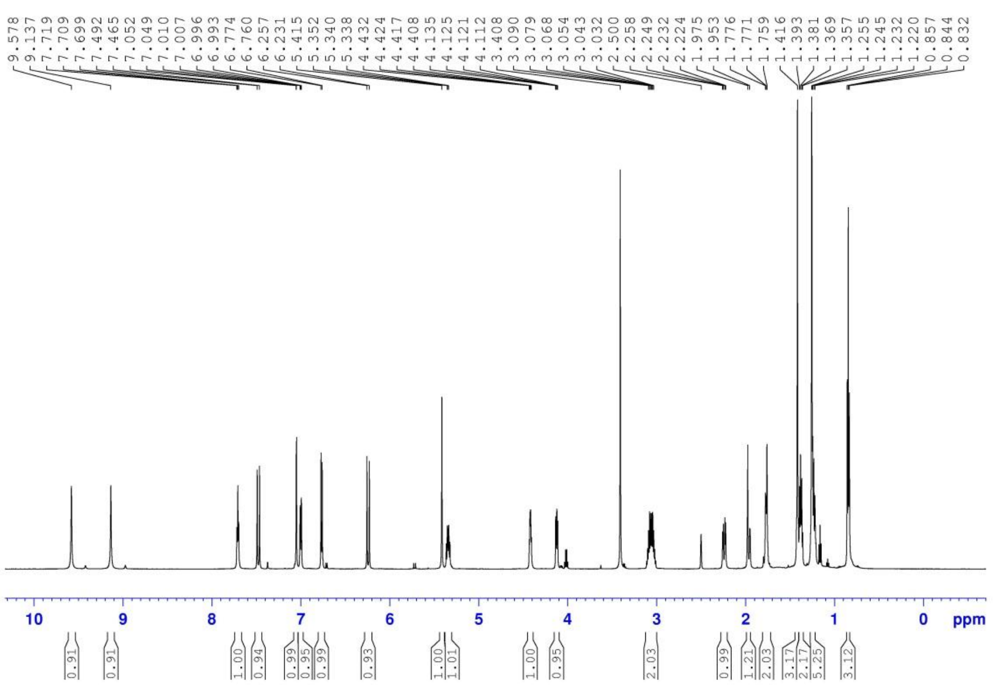

^{1}H NMR of compound **3b**

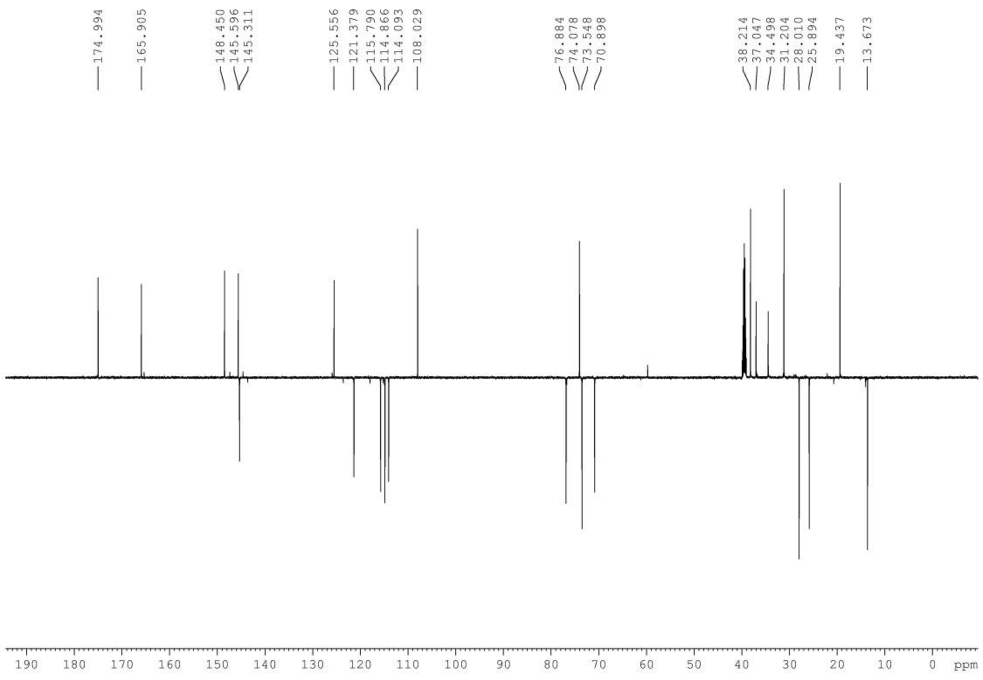

^{13}C NMR of compound **3b**

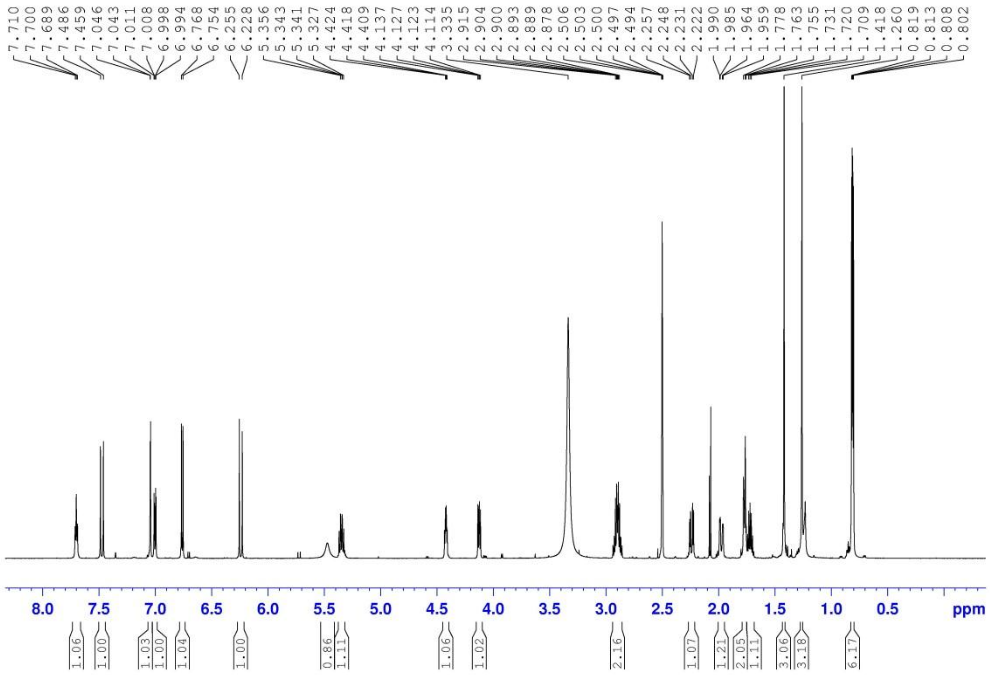

^{1}H NMR of compound **3c**

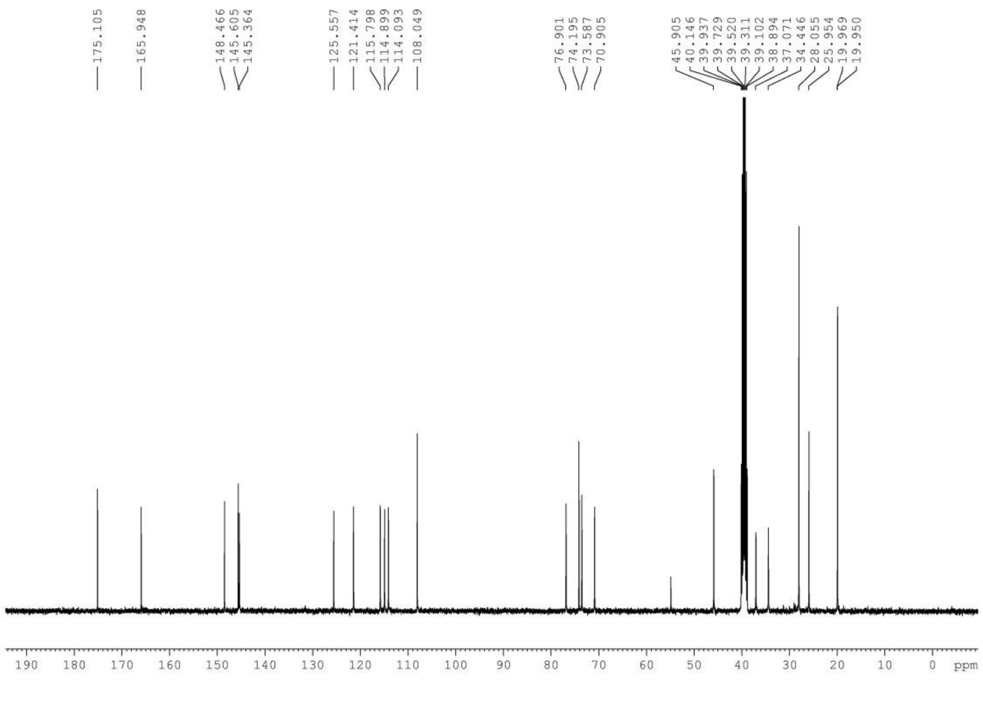

^{13}C NMR of compound **3c**

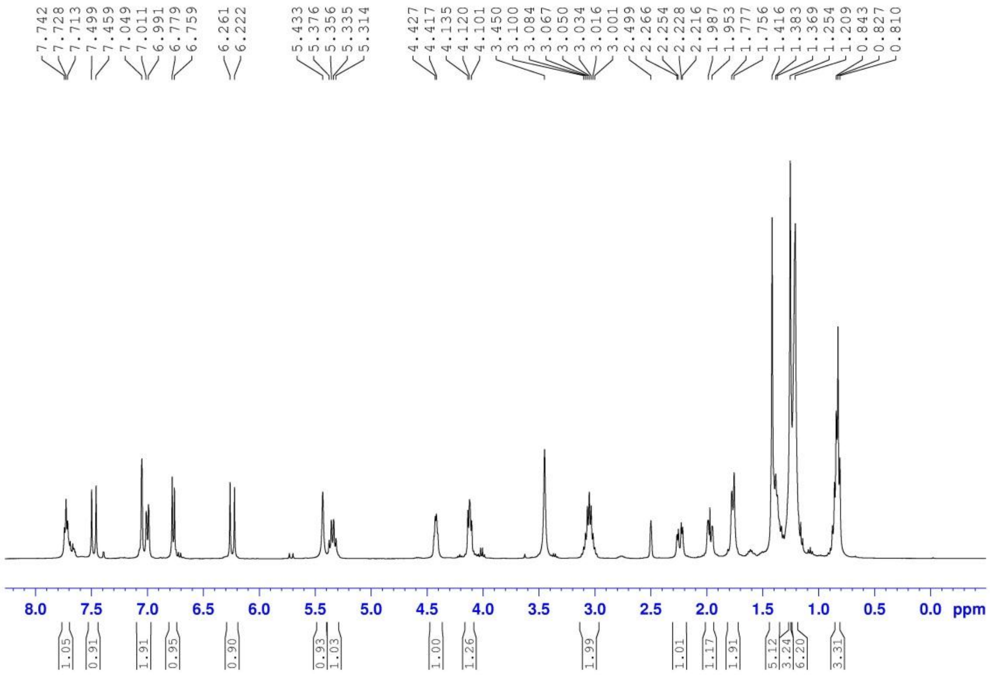

^{1}H NMR of compound **3d**

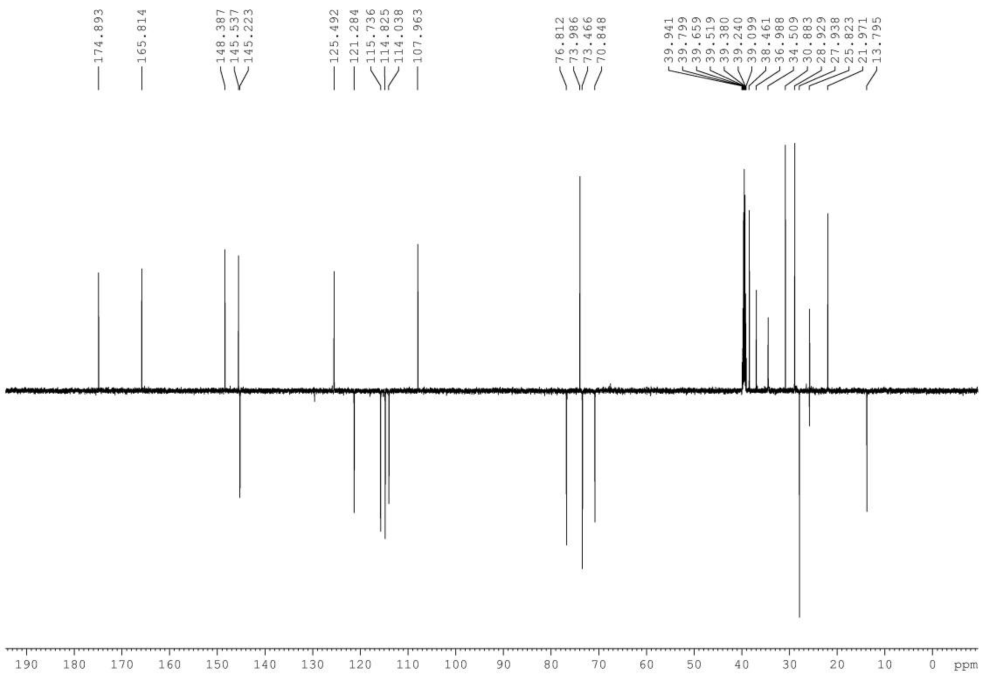

^{13}C NMR of compound **3d**

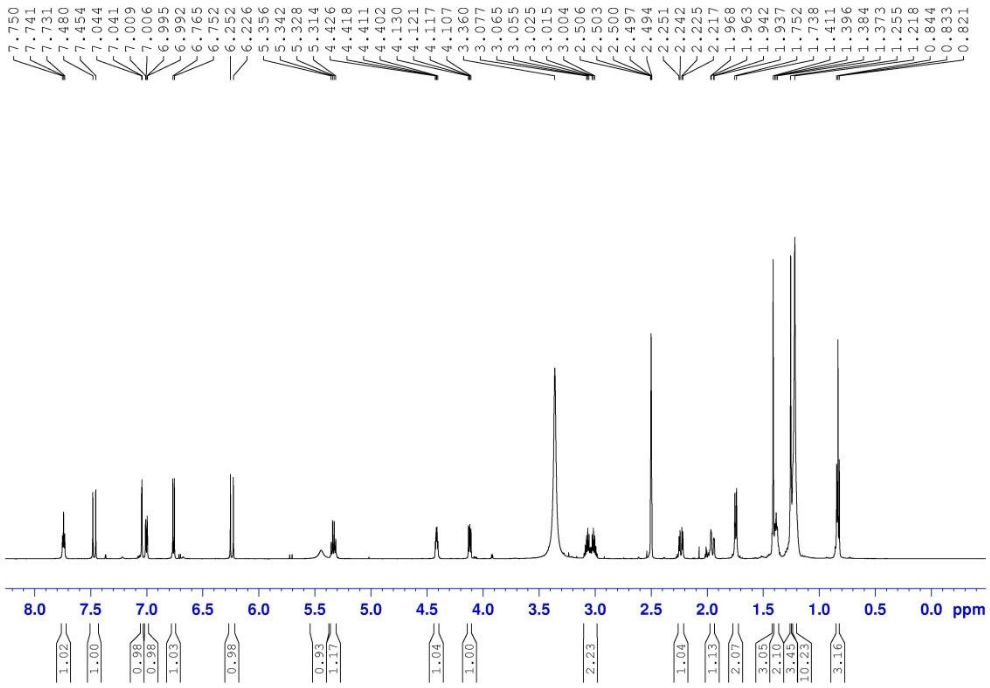

^{1}H NMR of compound **3e**

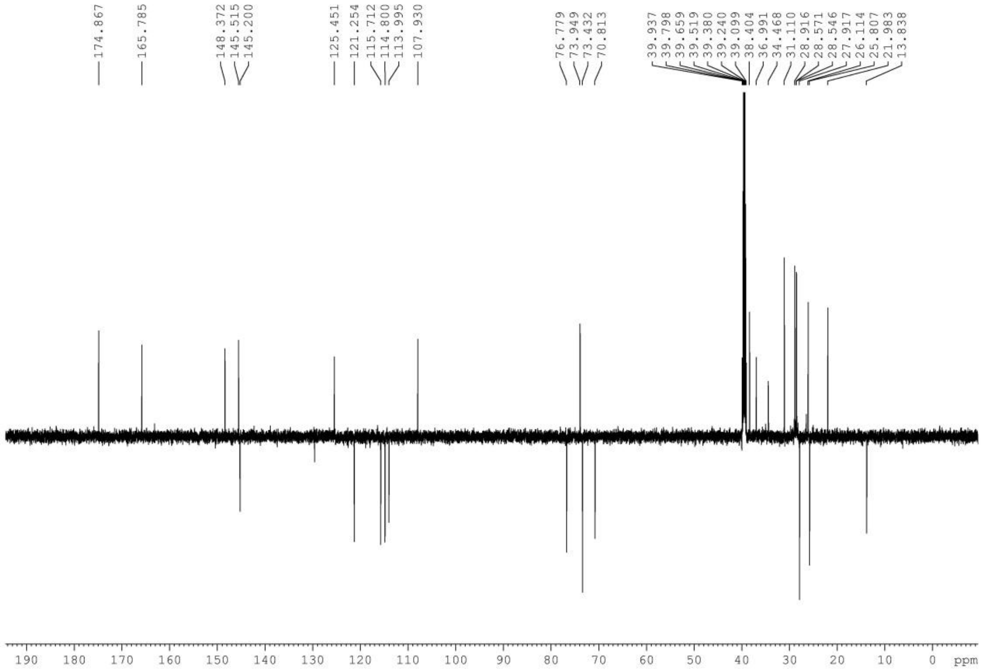

^{13}C NMR of compound **3e**

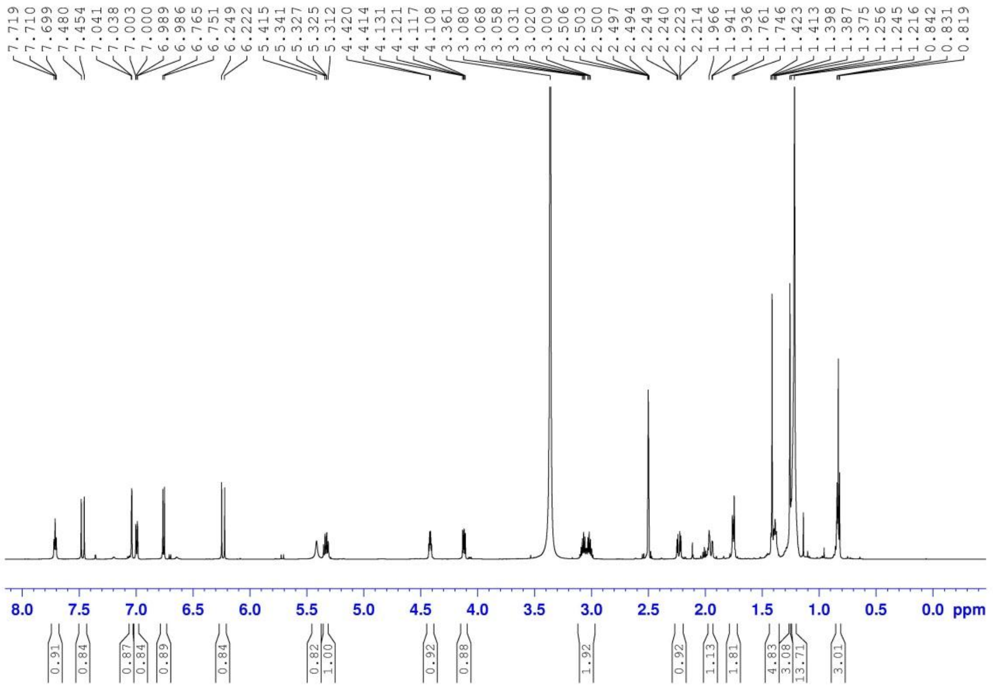

^{1}H NMR of compound **3f**

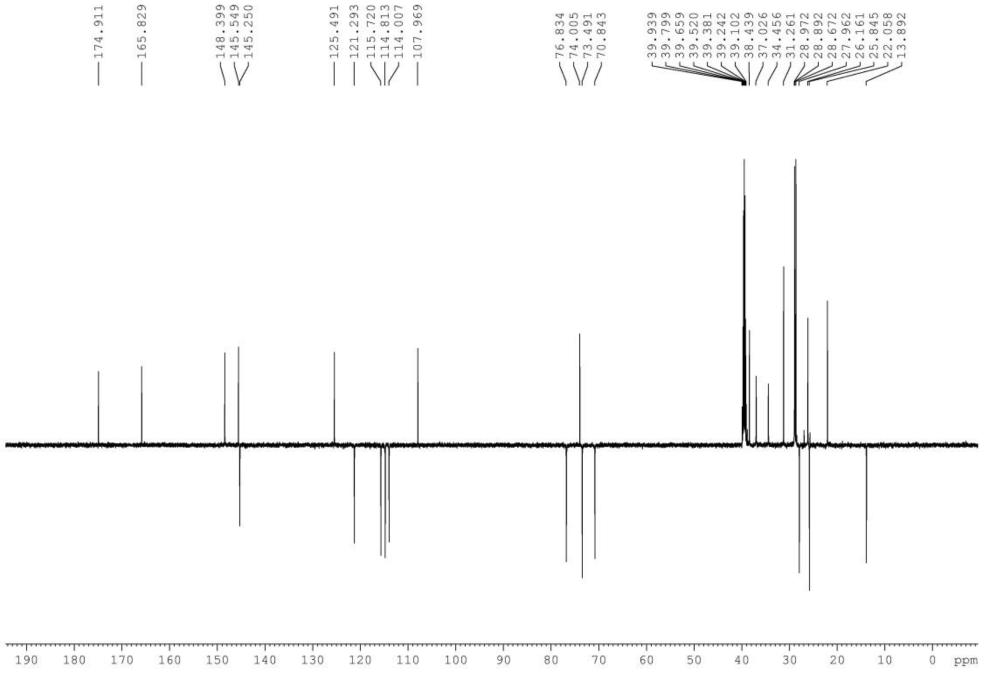

^{13}C NMR of compound **3f**

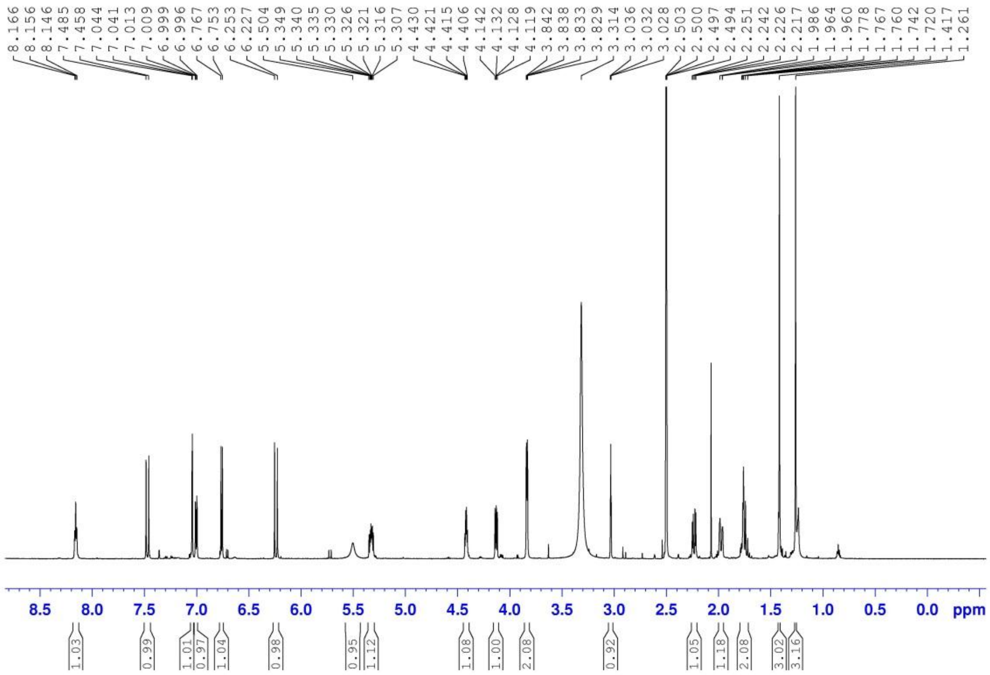

^{1}H NMR of compound **3g**

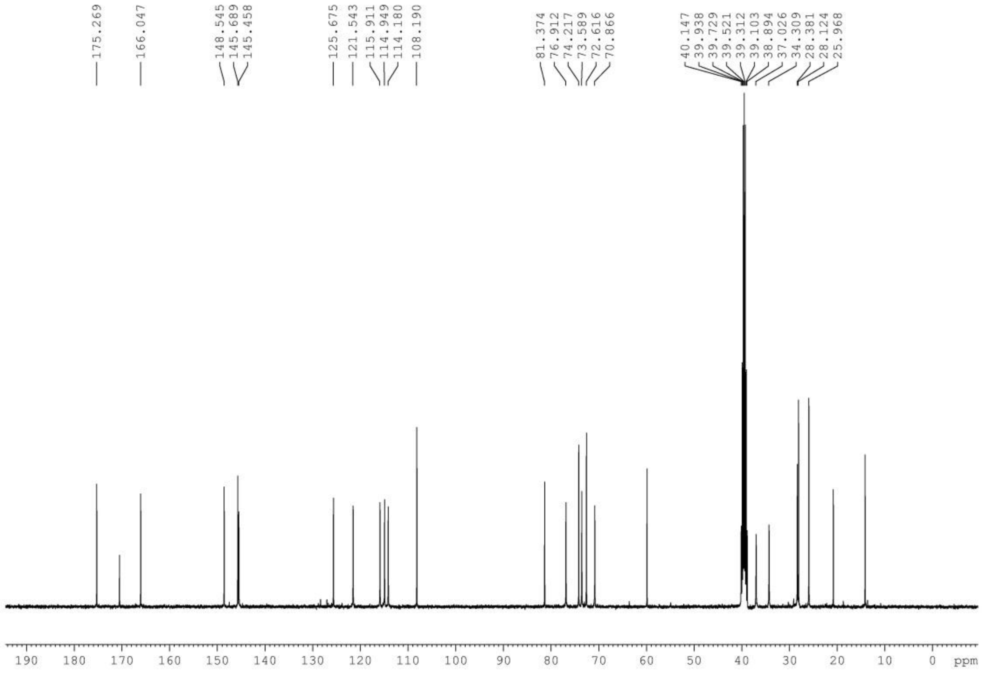

^{13}C NMR of compound **3g**

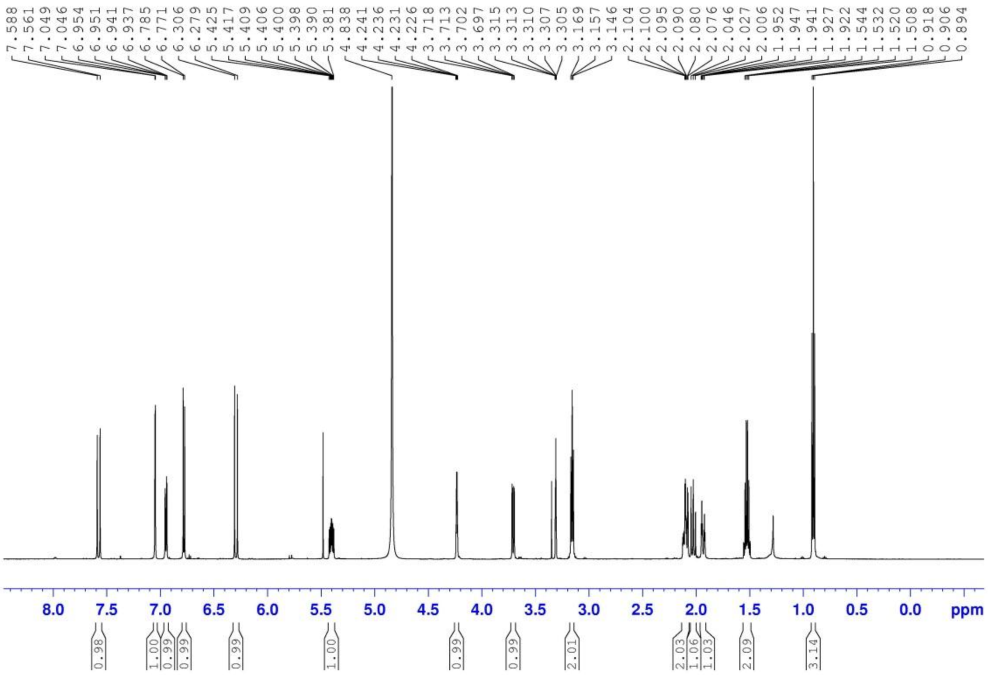

^{1}H NMR of compound **4a**

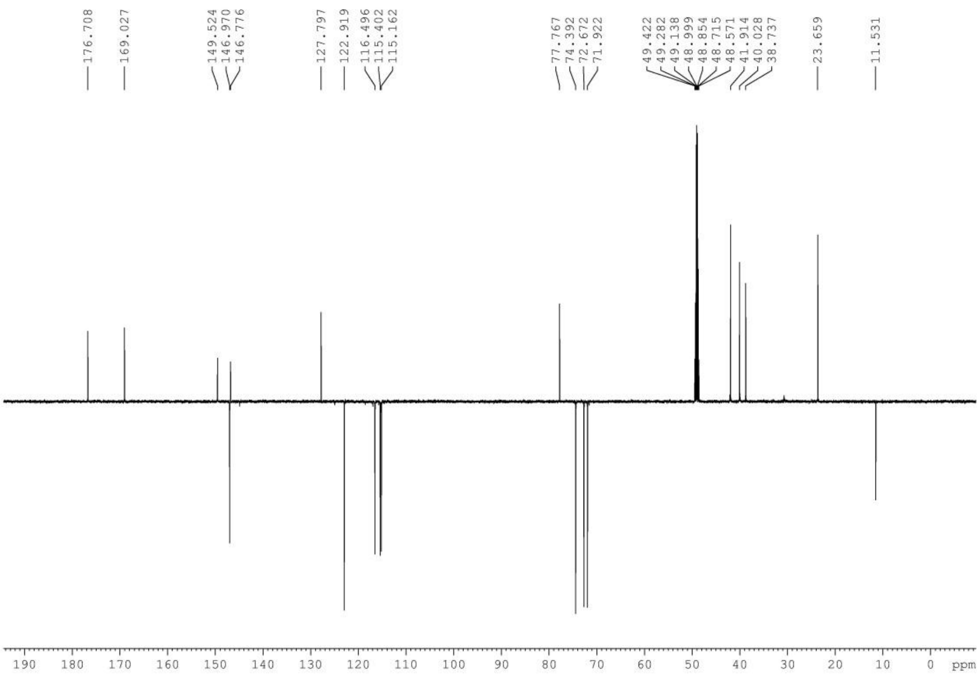

^{13}C NMR of compound **4a**

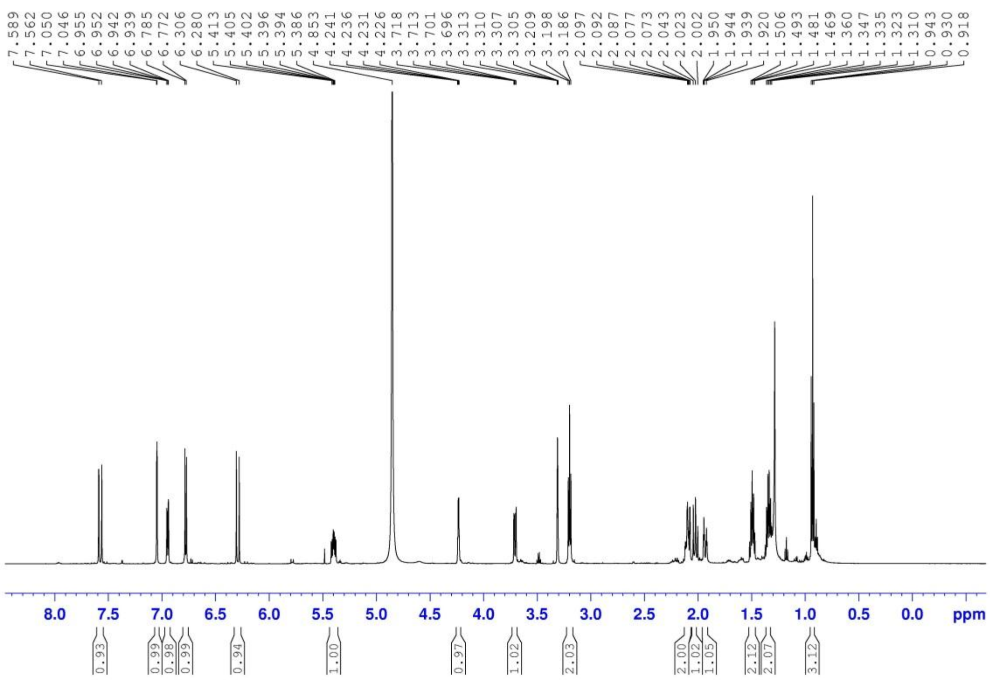

^{1}H NMR of compound **4b**

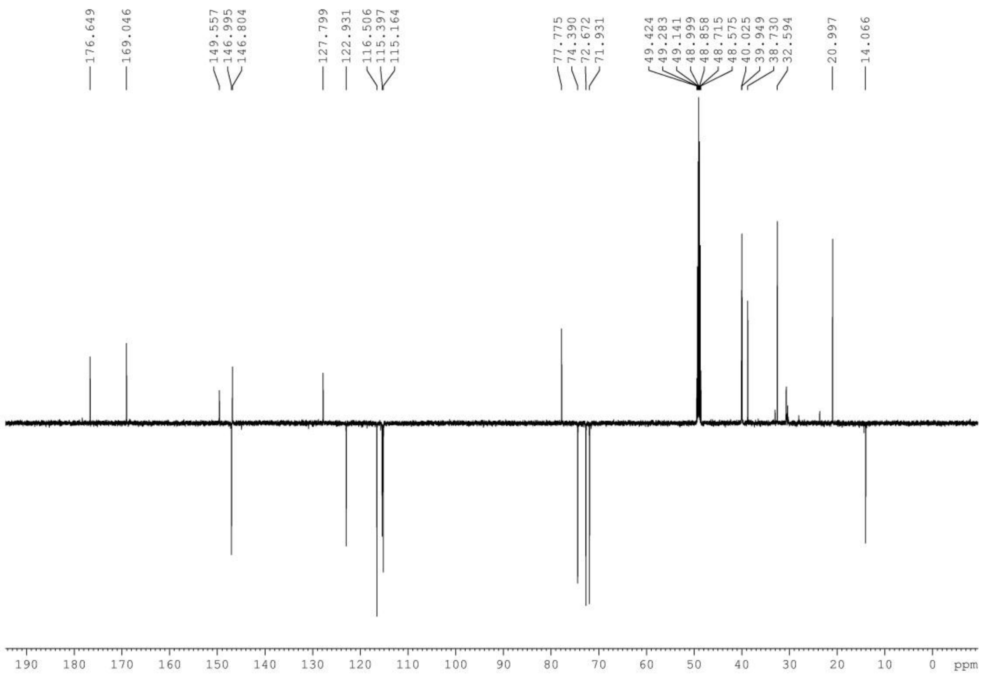

^{13}C NMR of compound **4b**

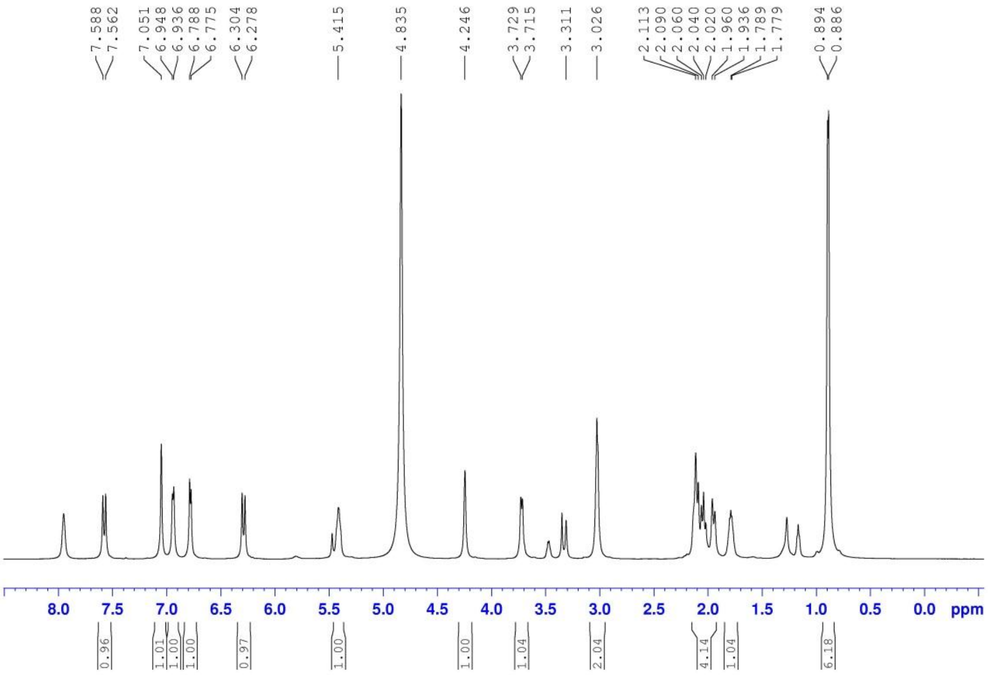

^{1}H NMR of compound **4c**

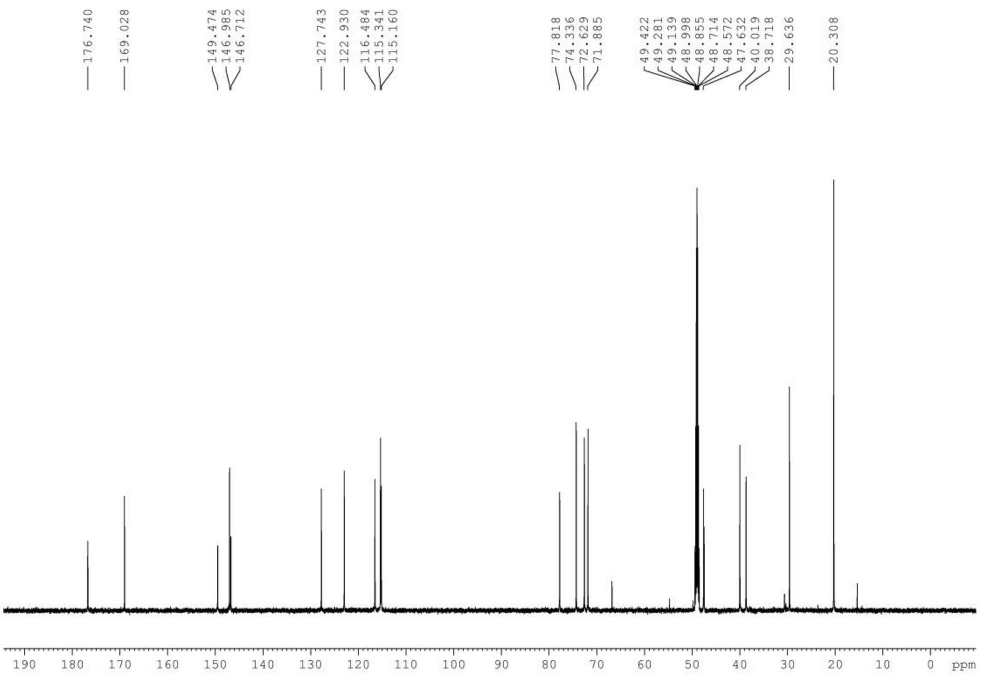

^{13}C NMR of compound **4c**

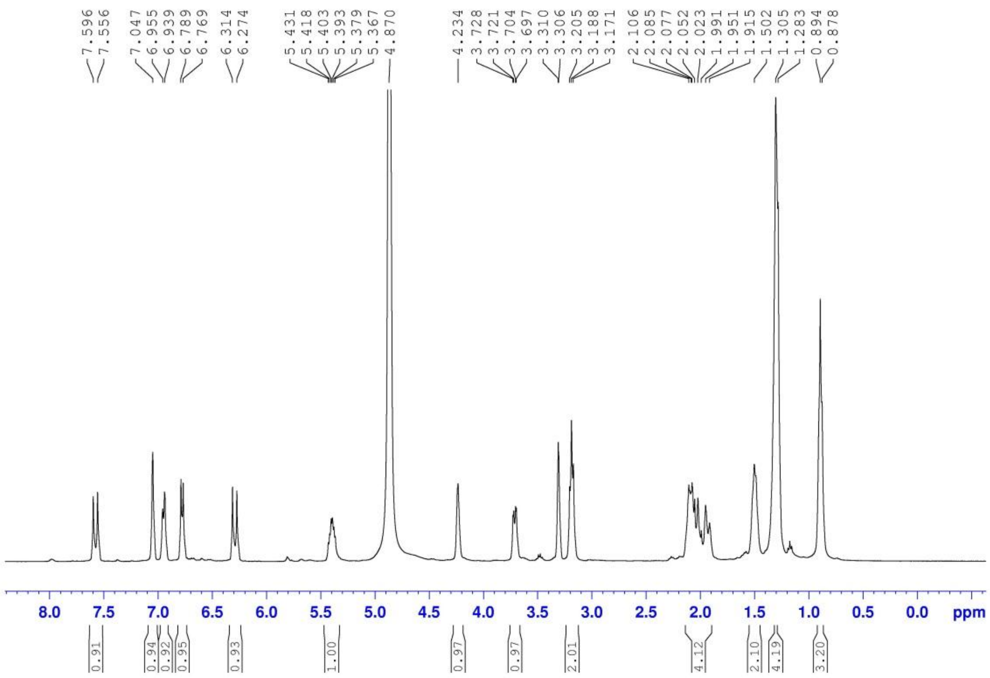

^{1}H NMR of compound **4d**

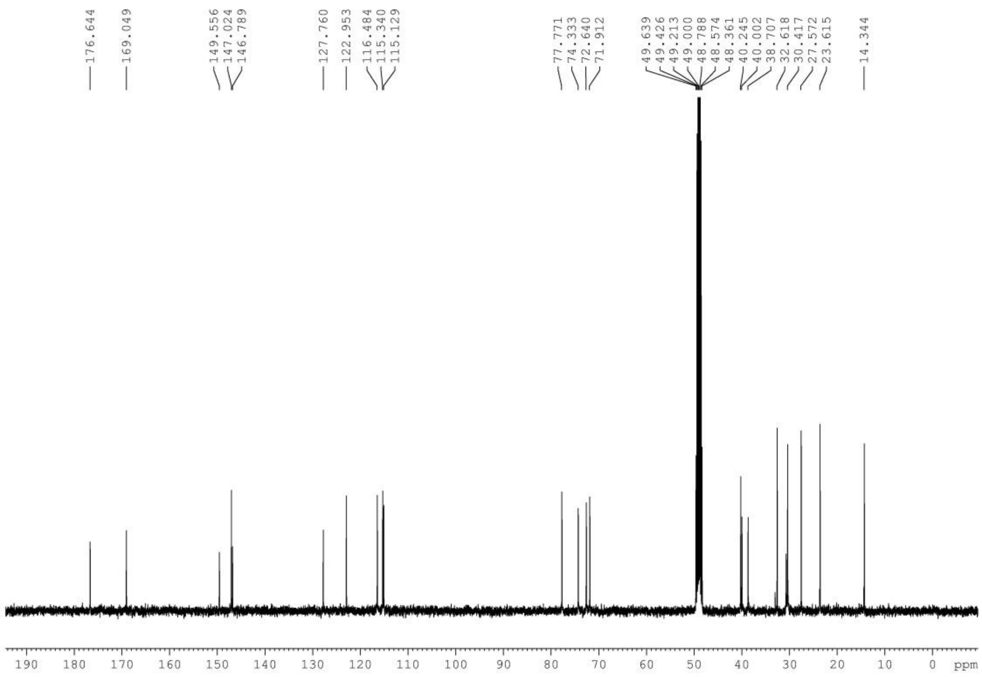

^{13}C NMR of compound **4d**

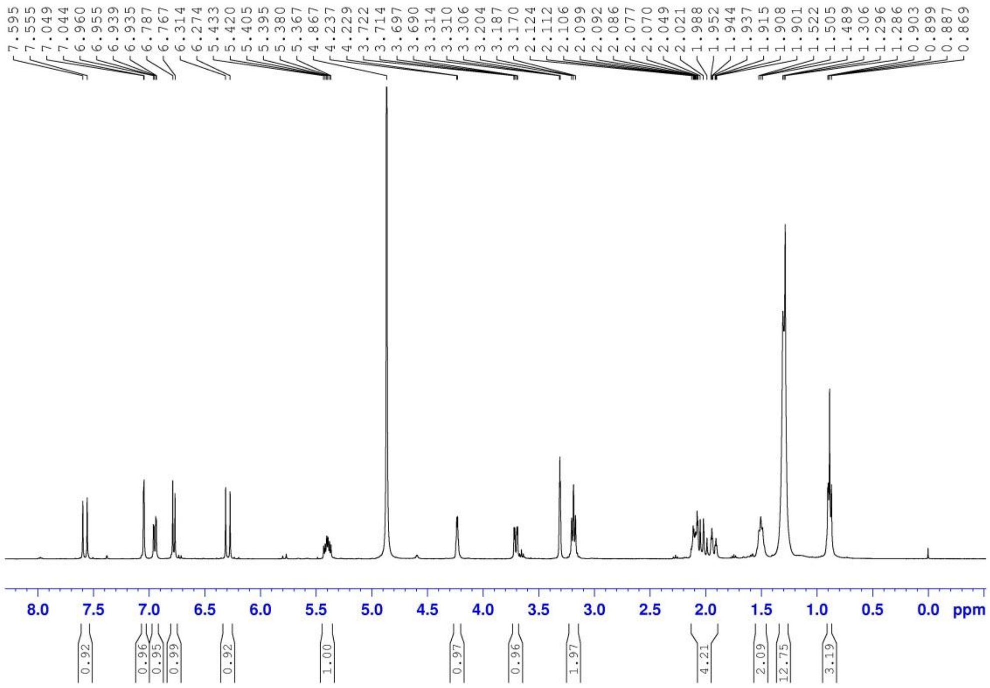

^{1}H NMR of compound **4e**

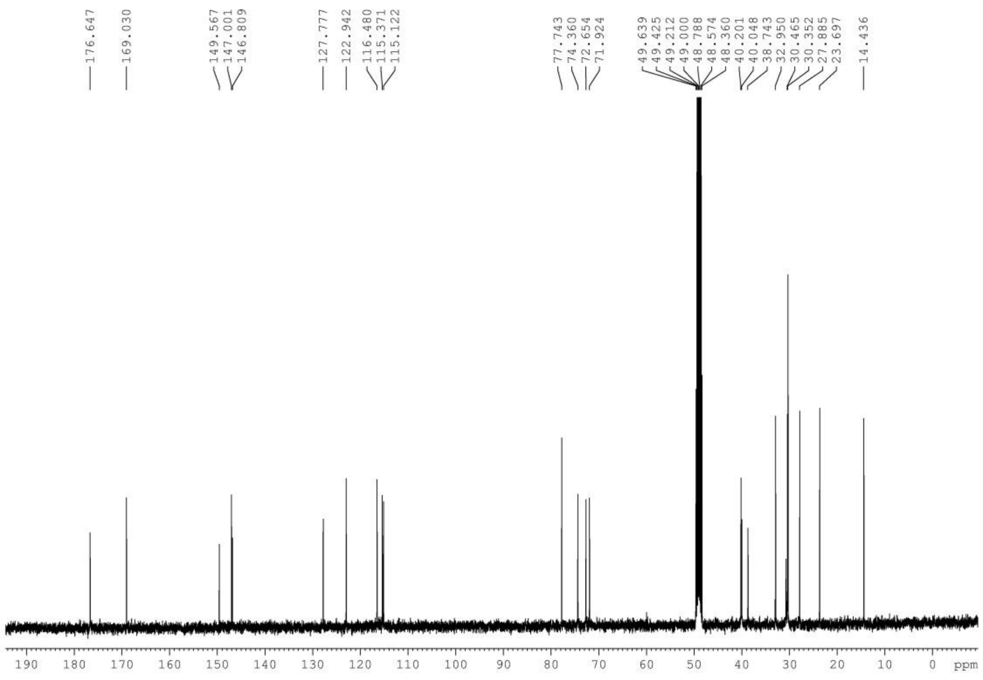

^{13}C NMR of compound **4e**

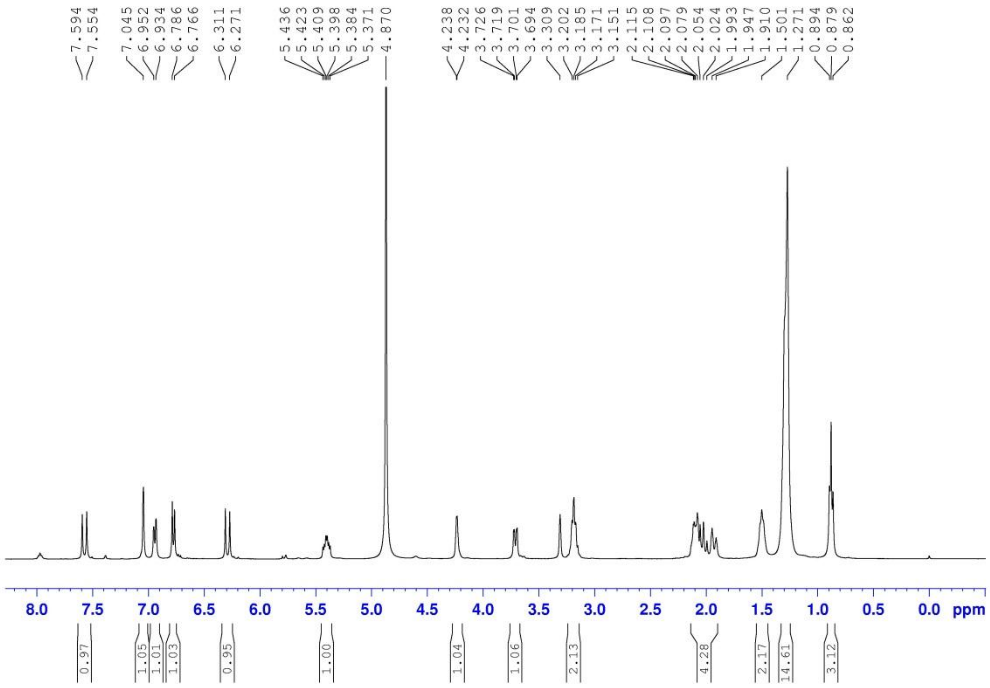

^{1}H NMR of compound **4f**

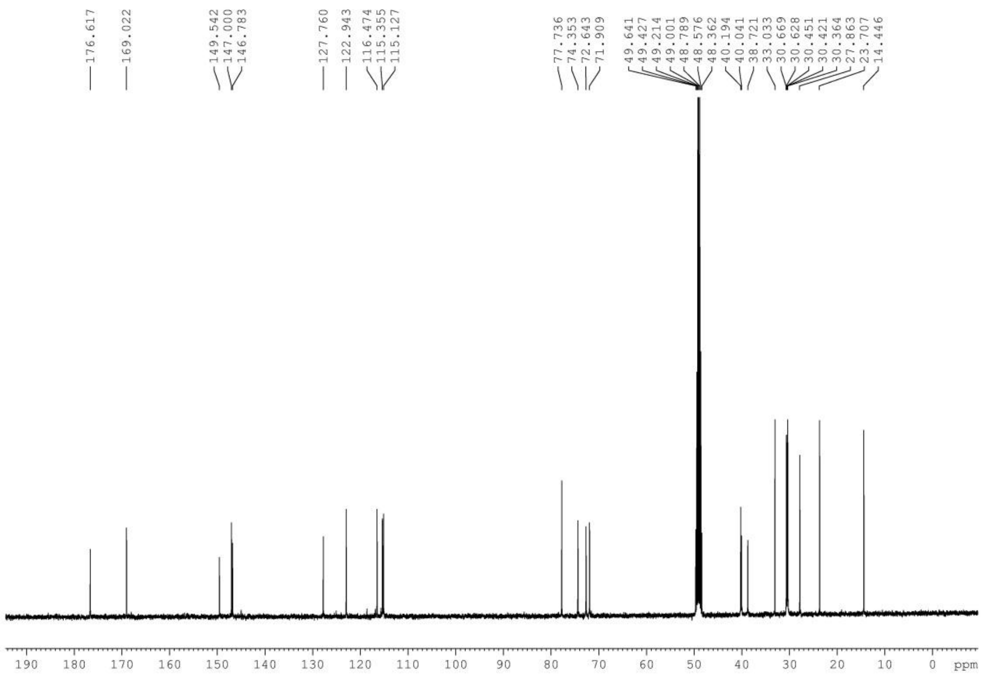

^{13}C NMR of compound **4f**

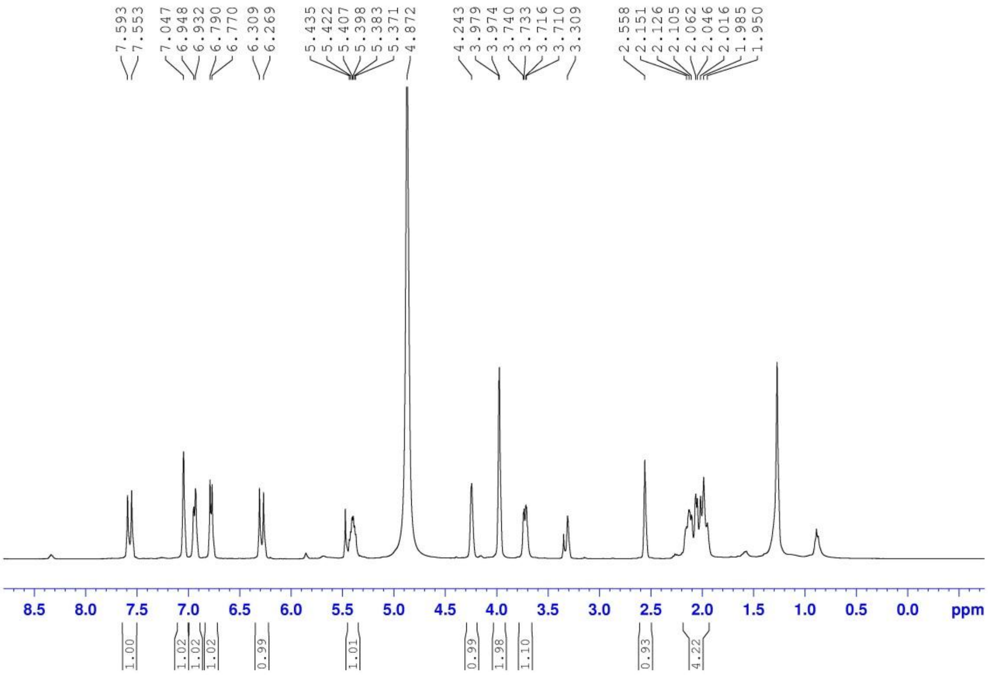

^{1}H NMR of compound **4g**

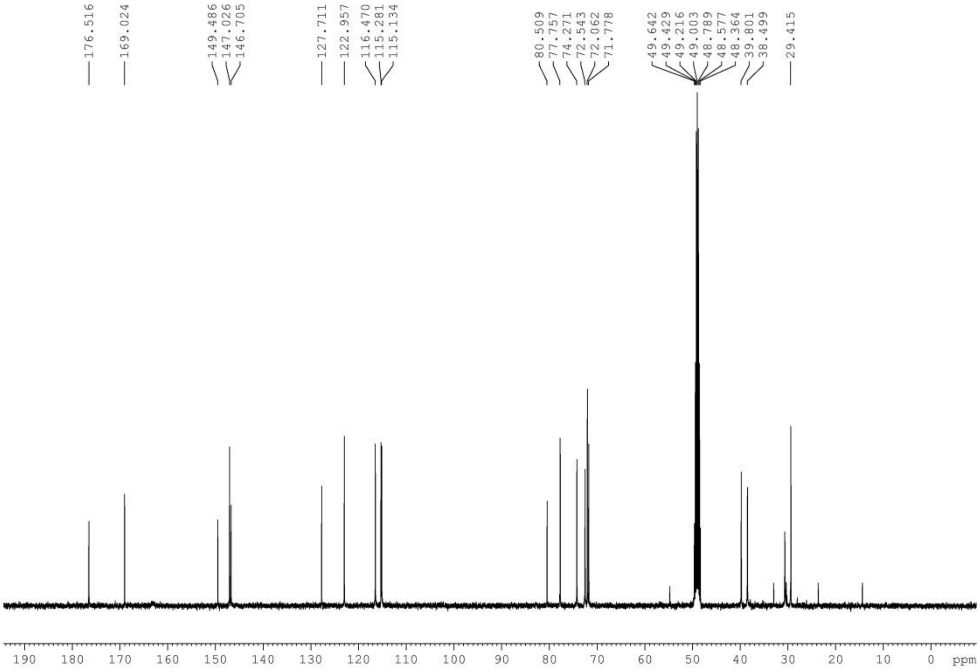

^{13}C NMR of compound **4g**

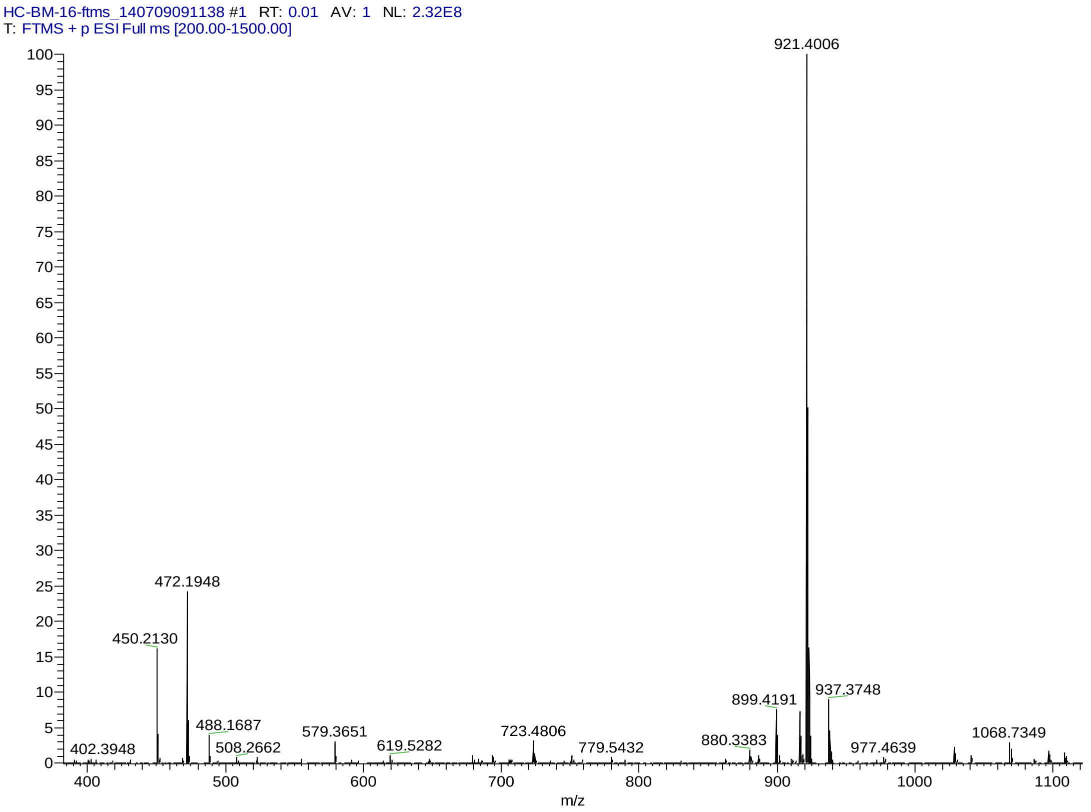

HRMS of compound **3a**

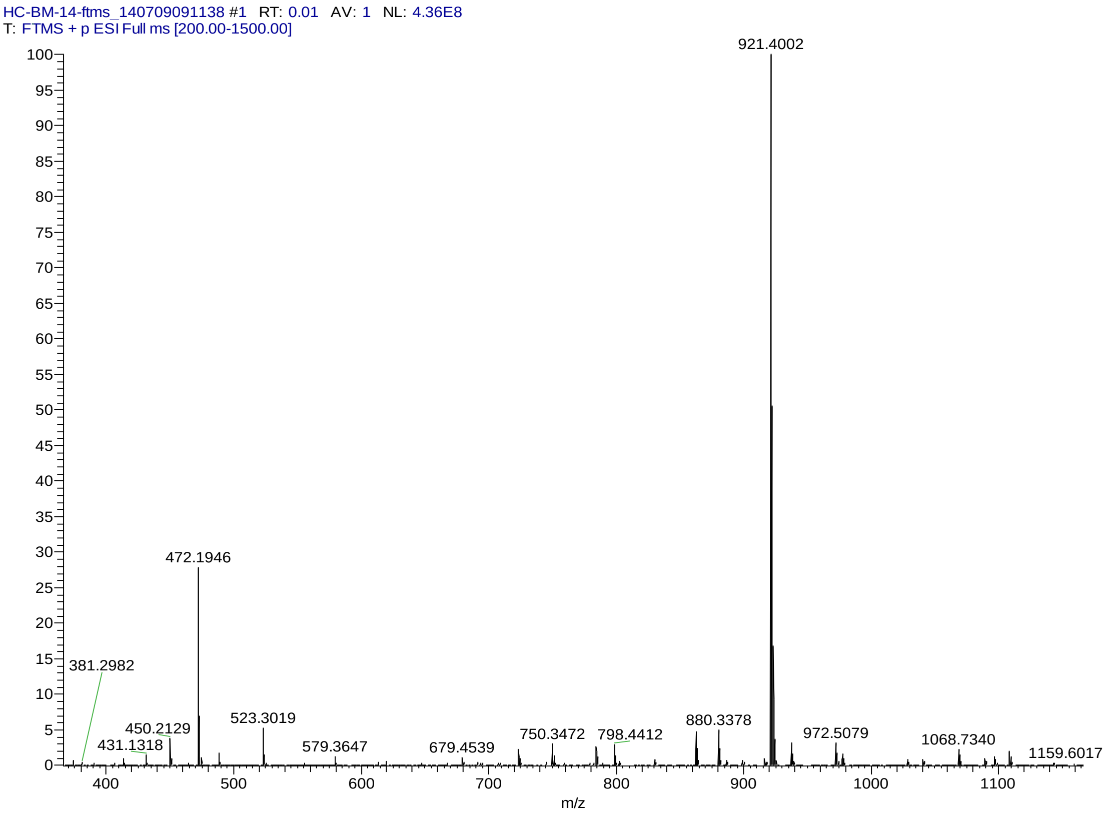

HRMS of compound **3b**

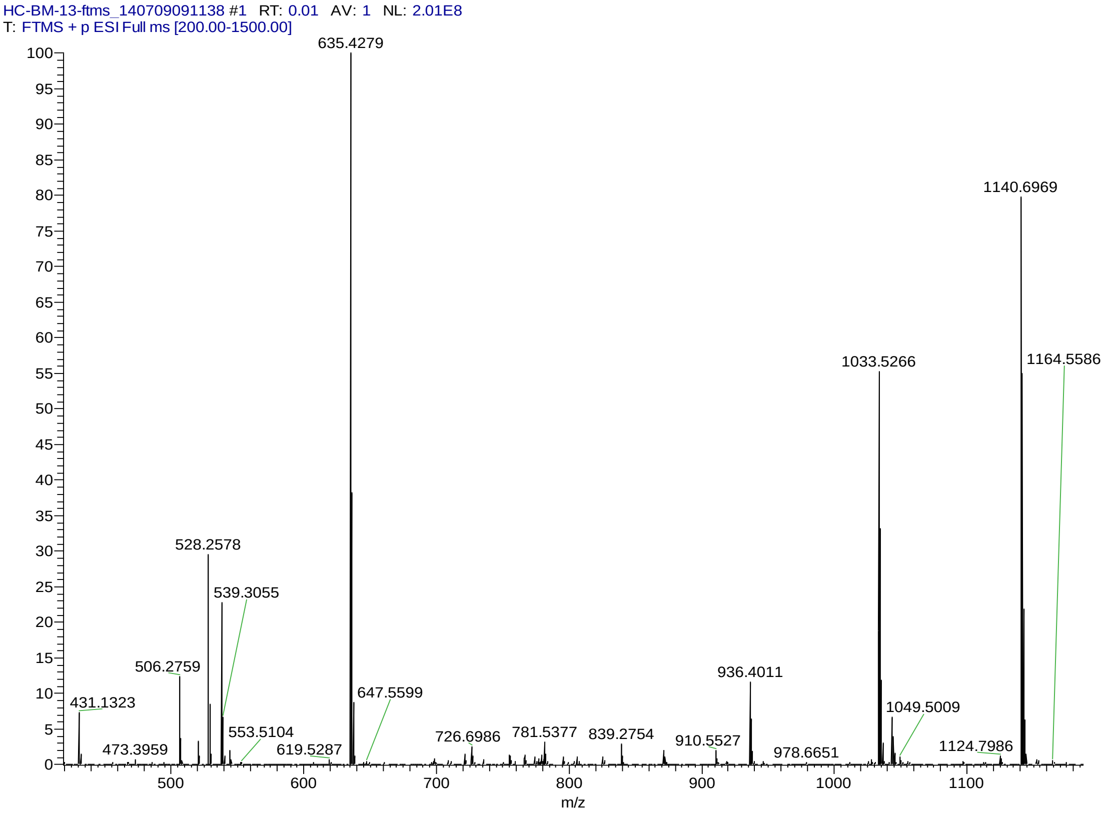

HRMS of compound **3c**

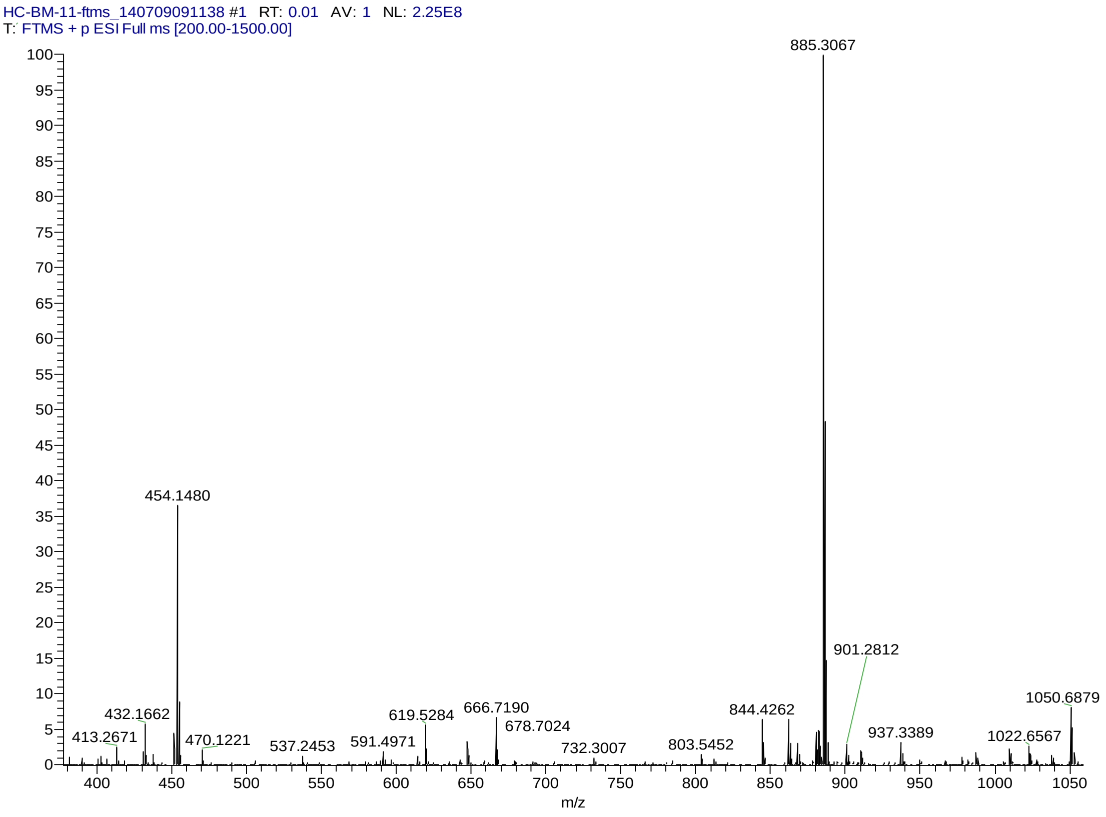

HRMS of compound **3d**

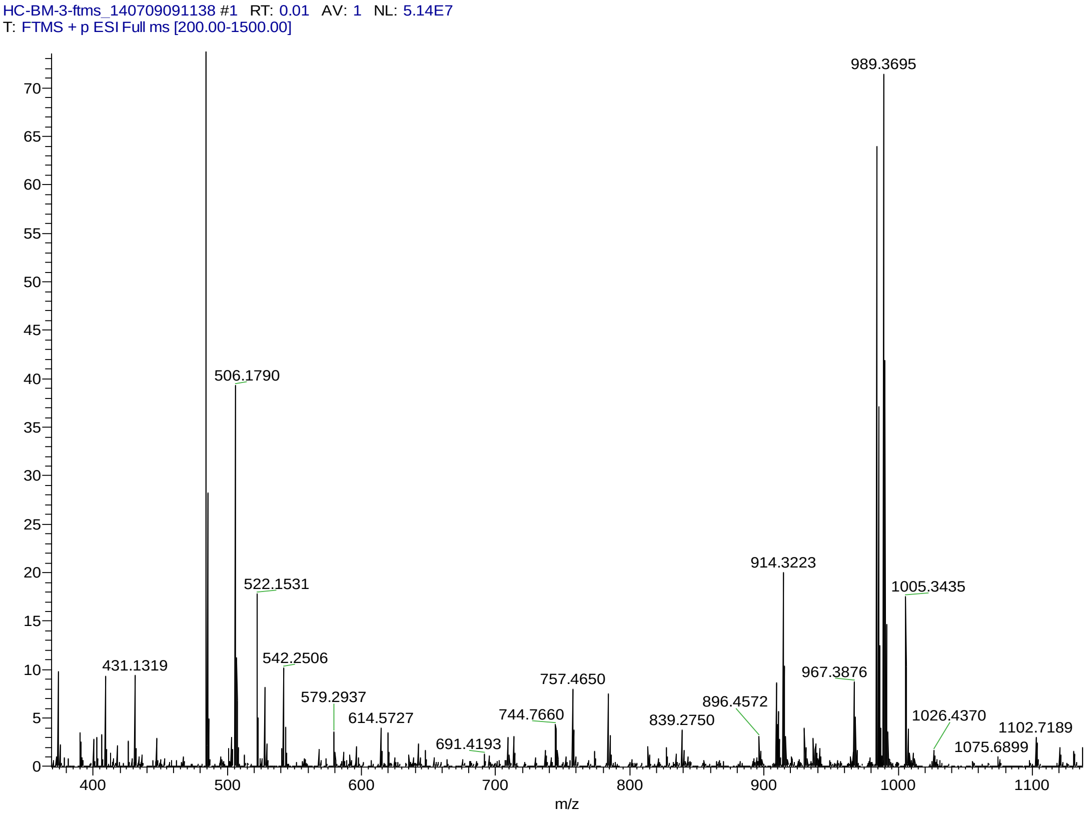

HRMS of compound **3e**

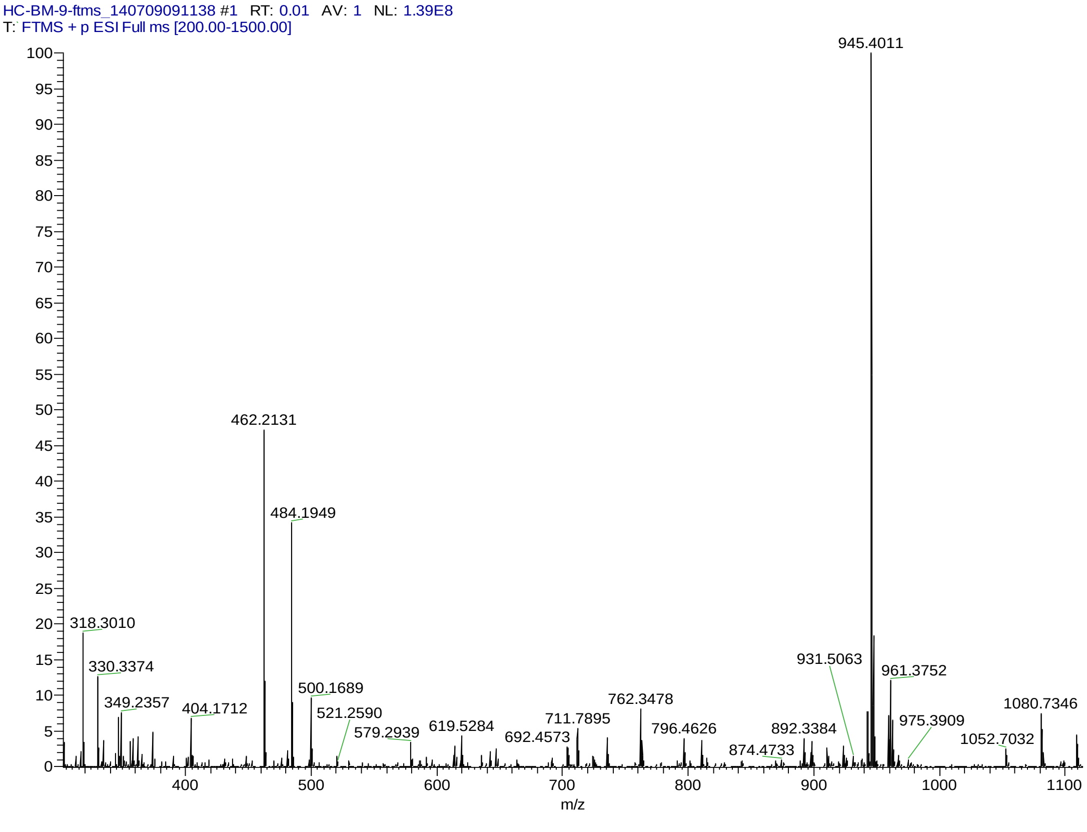

HRMS of compound **3f**

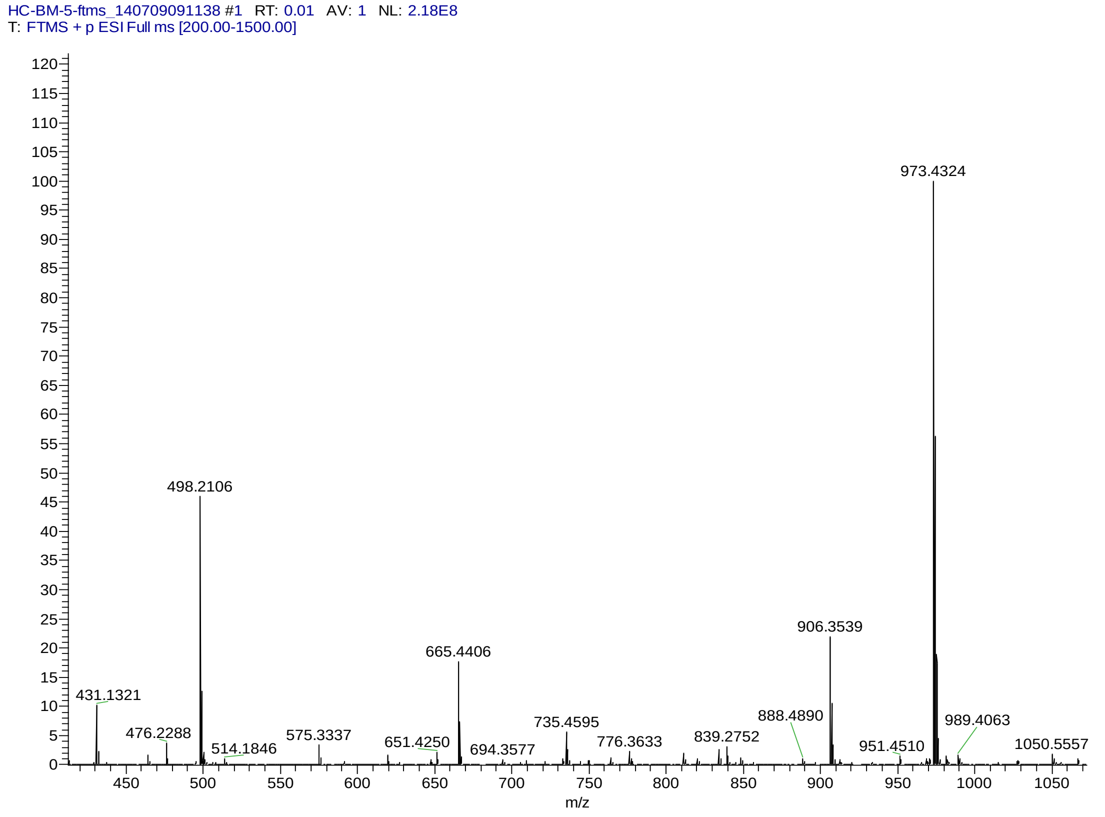

HRMS of compound **3g**

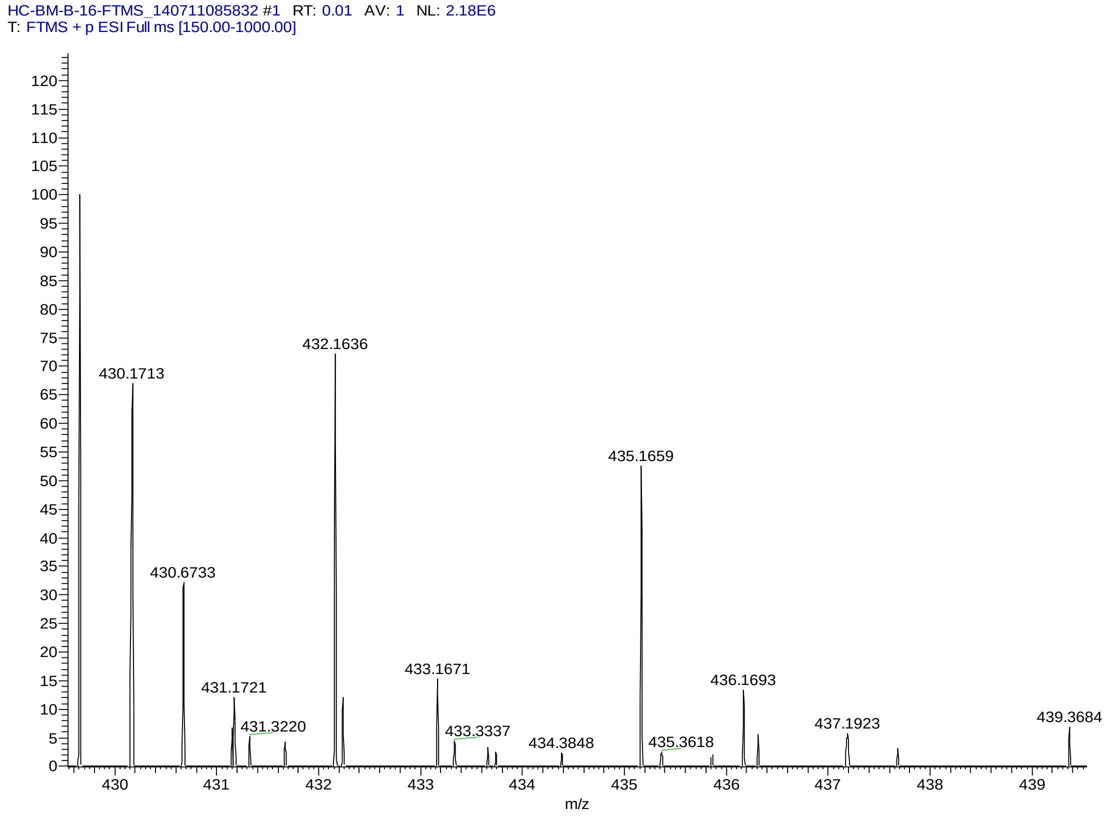

HRMS of compound **4a**

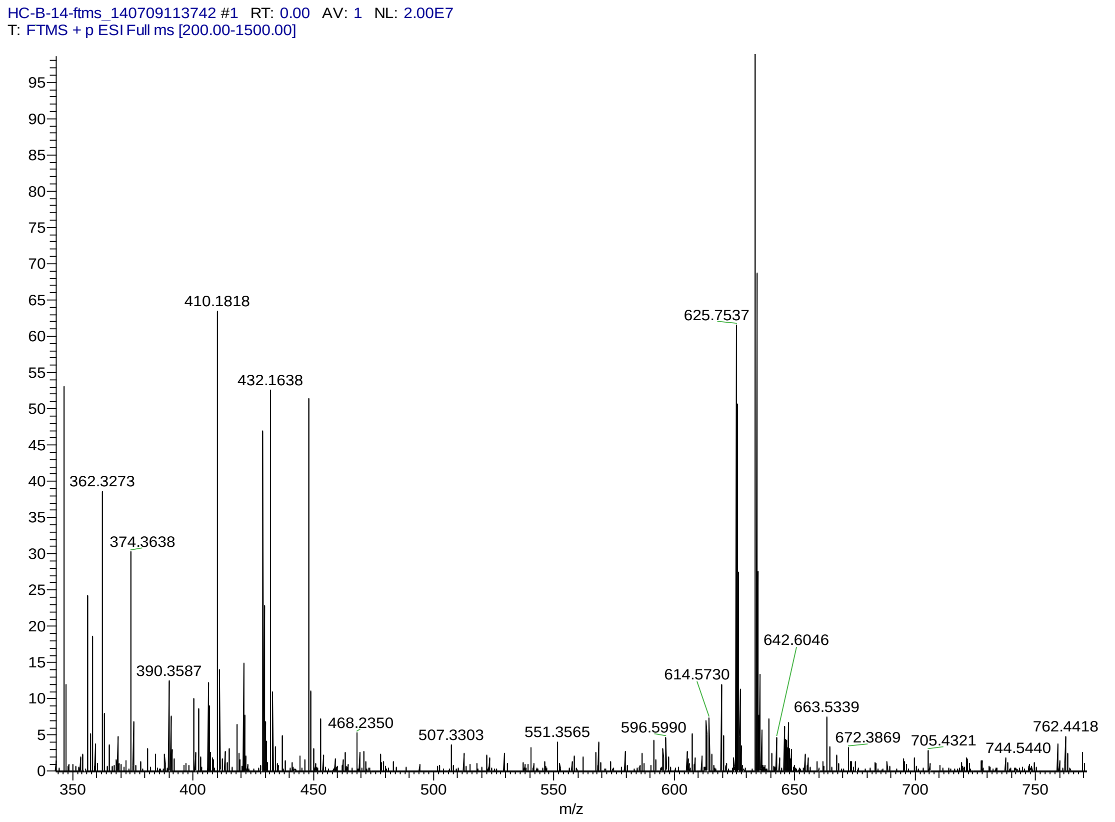

HRMS of compound **4b**

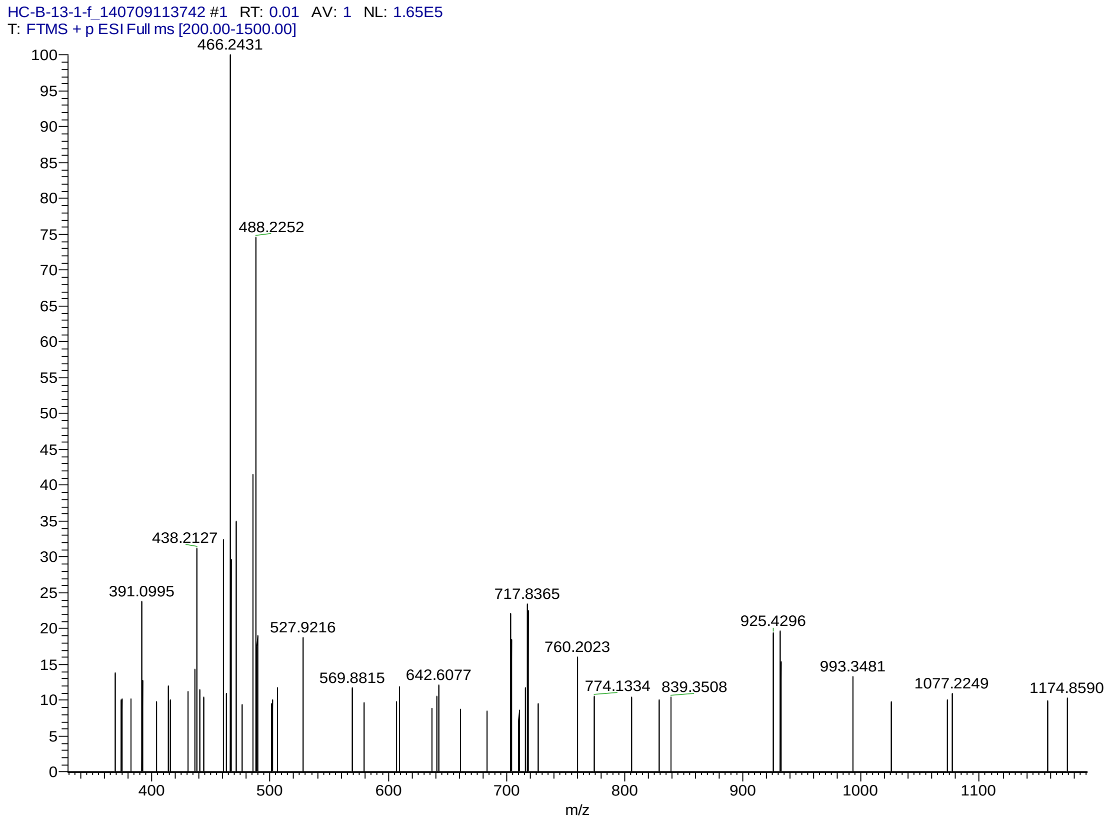

HRMS of compound **4c**

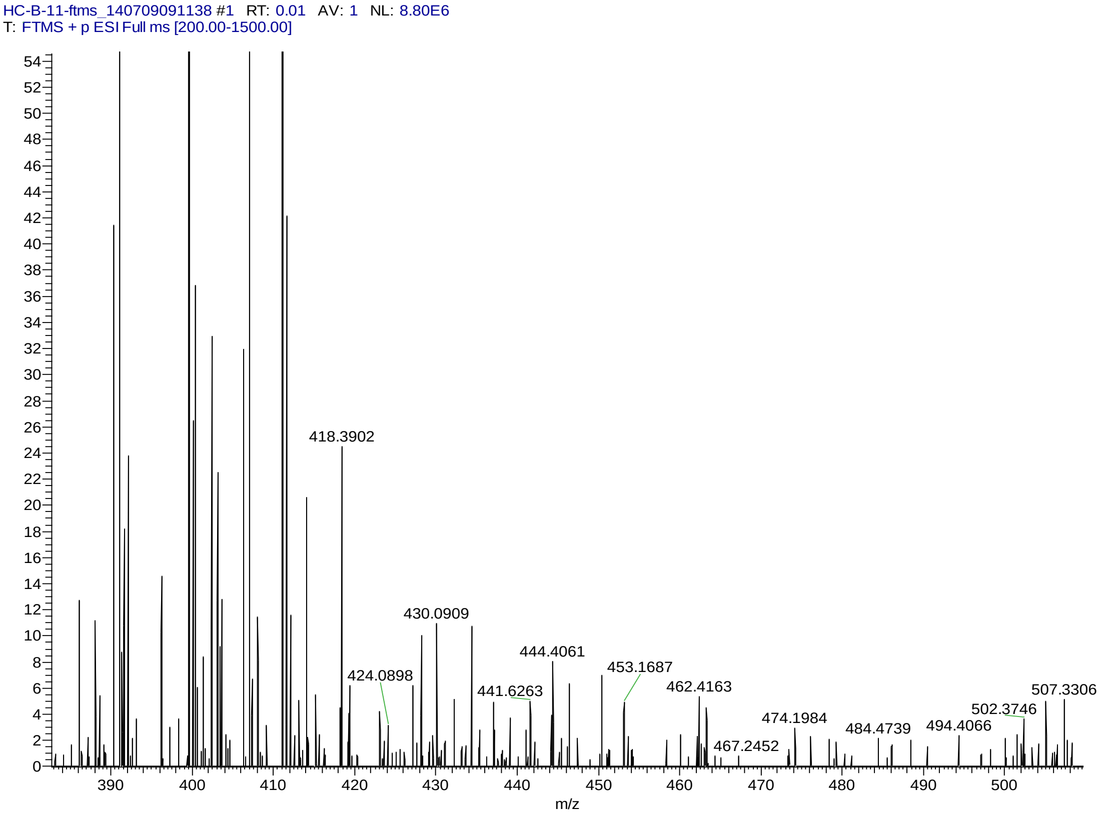

HRMS of compound **4d**

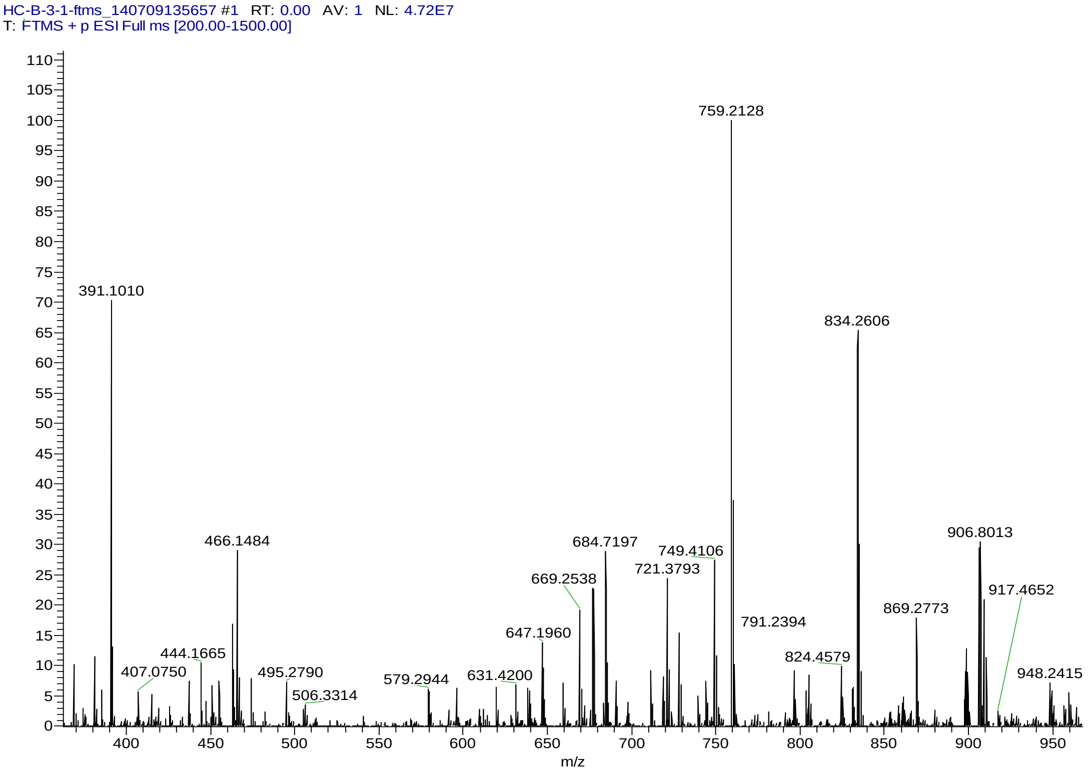

HRMS of compound **4e**

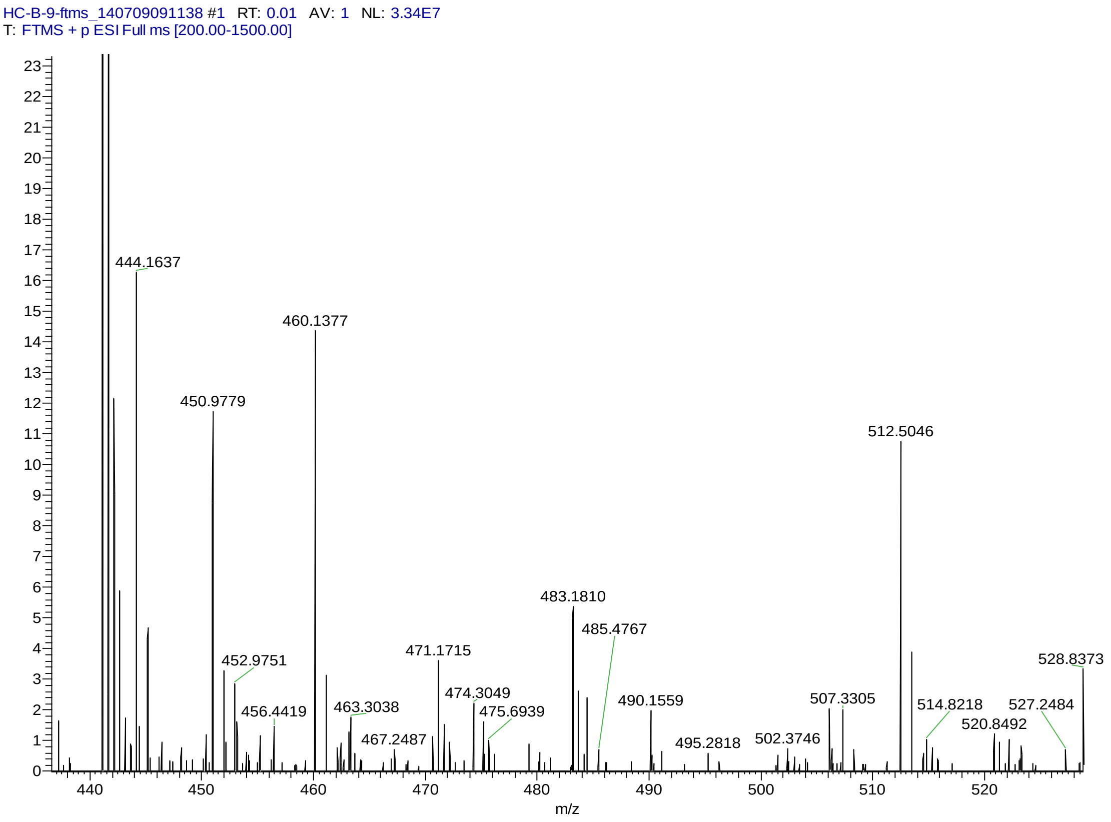

HRMS of compound **4f**

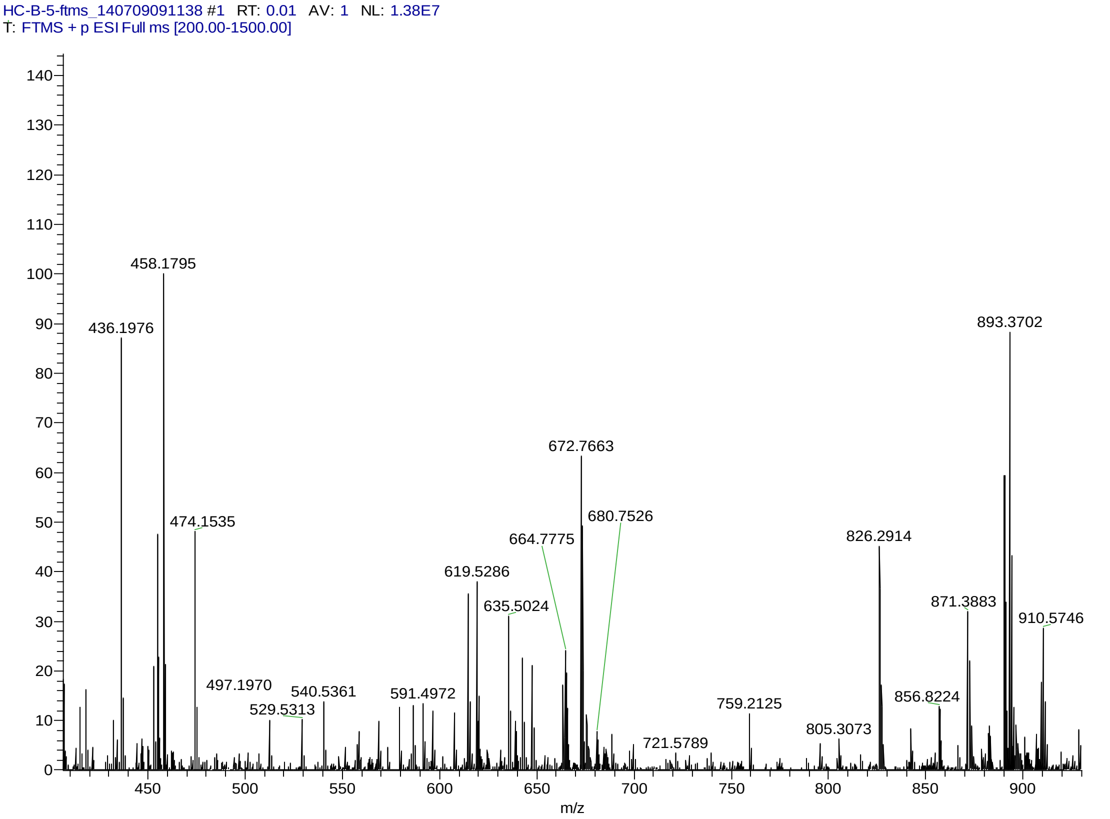

HRMS of compound **4g**
